# Photophysical Properties of Thionine and Methylene Blue Photosensitizers: A TD-DFT Study of Monomers, Salts, Hydrates, and Dimers

**DOI:** 10.3390/molecules31213842

**Published:** 2026-10-24

**Authors:** Christina Eleftheria Tzeliou, Eleni Dimou, Demeter Tzeli

**Affiliations:** 1 Laboratory of Physical Chemistry, Department of Chemistry, National and Kapodistrian University of Athens, Panepistimiopolis Zografou, 157 84 Athens, Greece; ctzeliou@chem.uoa.gr (C.E.T.); eldimou@chem.uoa.gr (E.D.); 2 Theoretical and Physical Chemistry Institute, National Hellenic Research Foundation, 48 Vassileos Constantinou Ave., 116 35 Athens, Greece

**Keywords:** photodynamic therapy, photosensitizers, thionine, Lauth’s violet, methylene blue, absorption emission, vibronic spectra, dimers, aggregations, salts, DFT calculations

## Abstract

Photodynamic therapy (PDT) is an established treatment in medicine that combines light, photosensitizers, and molecular oxygen to induce cell death. This work presents a theoretical investigation of the photophysical properties of two cationic photosensitizers for PDT, thionine (TH; Lauth’s violet) and methylene blue (MB), using DFT and TD-DFT. We systematically examine TH and MB cations, their chloride salts (TH-Cl, MB-Cl), monohydrated species (TH-Cl-W, MB-Cl-W), and homodimers [(TH-Cl-W)_2_, (MB-Cl-W)_2_] in water and benzene, along with the TH-Cl@W_61_ cluster to probe first-solvation-shell effects. All optimized geometries are stable. Absorption, emission and vibronic spectra are calculated. MB molecular systems show absorption maxima red-shifted by ~40 nm relative to TH analogs, while the principal visible dimer bands are blue-shifted by 22–37 nm compared to the corresponding monomers. Given that the present calculated MB and TH species present a T_1_ state lying in the 0.86–1.44 eV range, and that MB systems generally exhibit substantially larger oscillator strengths than TH and therefore more intense absorption, these results provide preliminary support for MB as a more promising photosensitizer than TH. Electronic absorption spectra are blue-shifted compared to the experimental ones, but vibronic coupling markedly improves agreement with experiment in the visible region, whereas UV peaks are described adequately without vibronic effects. Finally, the dimerization can offer a route to fine-tune photophysical properties, highlighting the relevance of small, aggregated chromophores for PDT, photocatalysis, and photon upconversion.

## 1. Introduction

Photodynamic therapy (PDT) is an increasingly established, minimally invasive treatment for certain precancerous and cancerous diseases, as well as for some non-cancerous diseases in dermatology, ophthalmology, and dentistry. It involves administering a photosensitizer, i.e., a light-sensitive drug, that preferentially accumulates in diseased tissue and then activating it with light of a specific wavelength. The activated photosensitizer generates reactive oxygen species (ROS) that kill target cells, with damage confined to the illuminated area, thereby sparing healthy tissue and reducing side effects typical of chemotherapy and radiation [1,2,3,4].

The basic mechanism of action is based on the theory of activation of a non-toxic photosensitizing compound by the appropriate light wavelength. Each photosensitizer needs to reliably activate at a high enough light wavelength useful for therapy. As in each group of drugs, a set of characteristics and conditions describing the ideal photosensitizer can be distinguished, such as a high degree of chemical purity, stability at room temperature, photosensitivity effect only in the presence of a specific wavelength, high photochemical reactivity, absorption minimum in the range from 400 nm to 600 nm, low dark toxicity and negligible cytotoxicity in the absence of light, easy solubility in body tissues, rapid clearance from the body post-procedure, natural fluorescence, and inexpensive and simple synthesis, along with easy availability [5,6,7]. The above combination of photosensitizer and light can cause the release of energy through the release of a singlet oxygen molecule and other oxygen derivatives that act directly and cytotoxically on tissues.

Given that the photosensitizer is the core component of PDT, its photophysical properties largely determine how effective, safe, and clinically useful the treatment can be [8]. Thus, the investigation and design of good photosensitizers is central to making PDT more effective, applicable to more indications, and competitive with standard cancer therapies [5,6,7,8].

**Figure 1:**
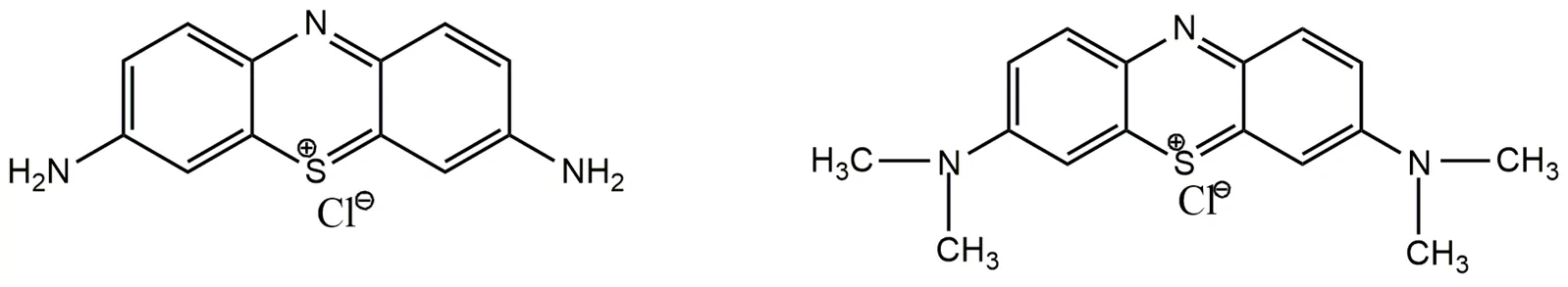
Structures of thionine (**left**) and methylene blue (**right**).

Thionine (3,7–diamino–5–phenothiazinium), commonly known as Lauth’s violet, is a cationic phenothiazinium dye with intriguing physicochemical features [9,10]. It was first synthesized by Charles Lauth (1836–1913) [11]; see Figure 1. When both amines are dimethylated, the resulting tetramethyl thionine is methylene blue, and the intermediates are Azure C (monomethyl thionine), Azure A (when one amine is dimethylated, while the other remains a primary amine), and Azure B (trimethyl thionine). In aqueous solution, the dye presents a concentration-dependent absorption spectrum due to the presence of aggregates. This aggregation is also influenced by pH. Experimentally, there are indications of dimerization of thionine and methylene blue dyes [12]. These dyes are able to transform into a colorless form through protonation from their triplet state and the attachment of electrons [13,14,15]. Furthermore, different forms of thionine exist in different media; for instance, at basic pH values, the free imine form of the thionine dye is formed, which is of a highly hydrophobic nature [16]. Thionine has been employed as a photosensitizer in photogalvanic studies, photodynamic therapy and as a photoinitiator of vinyl polymerization. This dye has a maximum of absorption around 600 nm, a high quantum yield of reactive oxygen species production and adequate solubility in aqueous medium [9,10]. Thionine is a common biological stain and is useful particularly in histology because of its high affinity for cellular membranes. As a member of the phenothiazinium family, it shows selectivity for tumor and microbial cells, which appears to be based on electrostatic interactions between the positive charge of these molecules and the negative charge found on the outer membrane/envelope of the target cell [17].

Methylthioninium chloride, commonly called methylene blue, is a heterocyclic aromatic chemical compound with the molecular formula of C_16_H_18_N_3_SCl. It is a formal derivative of phenothiazine. It is a dark green powder that yields a blue aqueous solution. Heinrich Caro first synthesized methylene blue in 1876; it has been reported as the first fully synthetic drug used in medicine, while a multitude of usages have been discovered amongst various fields of science [18]. The hydrated form has three molecules of water per unit of methylene blue [19]. The maximum light absorption is near 670 nm [12,13,14,15,20,21,22]. The specifics of absorption depend on a number of factors, including protonation, adsorption to other materials, and metachromasy—the formation of dimers and higher-order aggregates depending on concentration, solvent and other interactions [12,13,14,15,20]. This maximum lies well within the emission range of common diode lasers used for low-level laser therapy. Environmental light barely activates methylene blue. Therefore, adverse effects due to environmental light exposure are not to be expected. Moreover, methylene blue is a cost-effective photosensitizing agent that has been applied in antimicrobial photodynamic therapy (aPDT). Owing to its activity against a wide range of microorganisms, such as bacteria, fungi, and viruses, it has also been investigated as a locally administered PDT agent for the treatment of periodontal disease [23]. Finally, apart from PDT, methylene blue can present applications in photocatalysis [24,25], photon upconversion [26], photodegradation [27], etc.

The photophysical properties of thionine (TH) and methylene blue (MB) molecules or cations have been studied theoretically [28,29,30]. The electronic structure of thionine has been studied via DFT, MRCI and RI-CC2 methods [28], while recently, the vibronic absorption spectrum of thionine and methylene blue in an aqueous solution using Density Functional Theory (DFT) methodology has been studied with a series of functionals [29,30]. However, as far as we know, their salts, dimers and the effect of explicit water molecules on their calculation have not been studied before. In this work, the molecular structures and the photophysical properties of (i) the TH and MB cations, (ii) their salt with Cl^-^ (TH-Cl and MB-Cl), (iii) salts adding water molecules (TH-Cl-W and MB-Cl-W), and (iv) the corresponding homodimers [(TH-Cl-W)_2_ and (MB-Cl-W)_2_] have been studied theoretically in water solvent and, for some of them, in benzene solvent employing DFT. Additionally, the TH-Cl@W_61_ structure was calculated to examine the effect of the first sphere of solvation on the photophysical properties of TH-Cl. Linkage of monomers into dimers has been found to lead to fine-tuning of their photophysical properties, while dimeric chromophores, which are the smallest aggregated structures, can be used in fields including PDT, photocatalysis and photon upconversion. The effect of protonation and chlorination on their photophysical properties is also investigated.

## 2. Computational Details

Thionine (TH): The neutral thionine-water dimer (TH-W), the thionine cation with a water molecule (TH^+^-W), the chlorinated thionine (TH-Cl_H_, where the Cl^-^ anion has been attached close to a H atom of the TH^+^ cation), the chlorinated thionine (TH-Cl_S_, where the Cl^-^ anion has been attached close to the S atom of TH^+^), and the chlorinated thionine with a water molecule (TH-Cl-W) minimum structures have been calculated using DFT and Time-Dependent DFT (TD-DFT). The calculations were carried out using the B3LYP [31,32] functional in conjunction with the 6-31G(d,p) [33] and def2-TZVP [34,35] basis sets. To check the DFT methodology, the TH-Cl-W molecule has been calculated additionally with PBE0 [36,37], ωB97XD [38] and M06-2X [39] in conjunction with the def2-TZVP and 6-31G(d,p) basis sets. The ωB97XD functional includes an empirical dispersion correction and can therefore describe noncovalent interactions, including van der Waals interactions, more accurately [38]. In all calculations, the water solvent has been included explicitly and implicitly as a dielectric constant employing the polarizable continuum model (PCM) [40,41]. For all calculated structures, frequency calculations were performed to verify that the optimized structures are true minimum structures.

Additionally, the dimer of thionine with Cl^-^ has been calculated. Specifically, the two dimers (TH-Cl-W)_2_ that differ in the relative position of the Cl^-^ anions have been calculated via DFT using the B3LYP and ωB97XD functionals in conjunction with the def2-TZVP and 6-31G(d,p) basis sets in water solvent. Furthermore, the molecular system TH-Cl_S_@W_61,_ where the TH-Cl_S_ molecule is surrounded by 61 molecules of water, has been calculated at the ONIOM (B3LYP/6-31G(d,p): PM6) methodology [42], The system is defined as two regions (layers), where the higher layer is the TH-Cl_S_ molecule, which is calculated via DFT, while the lower layer consists of the 61 water molecules which are calculated at the semi-empirical PM6 method [43]. Note that the ONIOM methodology has been proven to predict the geometries of the system well [42,44] and the electronic spectra of the molecule of the higher layer calculated via DFT [45,46].

Methylene Blue (MB): The MB-Cl_H_ and MB-Cl_S_ minimum structures where a Cl^-^ anion has been attached close to a hydrogen atom and to a sulfur atom, respectively, have been calculated. Additionally, the MB-Cl-W minimum structure and the (MB-Cl-W)_2_ dimer of methylene blue with Cl^-^ has been calculated, which is part of the chlorinated methylene blue. The calculations were performed at the B3LYP and ωB97XD/def2-TZVP levels of theory in water solvent, which has been included implicitly as a dielectric constant. For all calculated structures, frequency calculations were performed to verify that the optimized structures are true minimum structures.

In all cases, the absorption spectra of the structures were obtained, including up to 40 singlet excited states in water solvent. Also, the absorption spectra in the case of MB in benzene solvent have been calculated. Generally, B3LYP and PBE0 usually predict the absorption spectra well [47,48,49,50]. However, all present functionals used have been applied to investigate their applicability. Furthermore, the linear response correction (cLR) approach has been applied to the main absorption peaks [51,52] to investigate its effect on the calculation of the absorption electronic spectra.

Finally, the vibronic absorption spectra were also calculated at the B3LYP/6-31G(d,p) level. The ground-state geometry was optimized, followed by a harmonic frequency calculation to confirm that the structure corresponded to a minimum. The geometry and vibrational frequencies of the relevant excited state were also obtained. The vibronic spectrum was then simulated within the Franck–Condon, Herzberg–Teller approximation [53,54]. The calculated vibronic transition energies and intensities were used to generate the simulated absorption spectrum. All calculations have been performed using the Gaussian 16 program [55].

## 3. Results

### 3.1. Thionine

**Figure 2:**
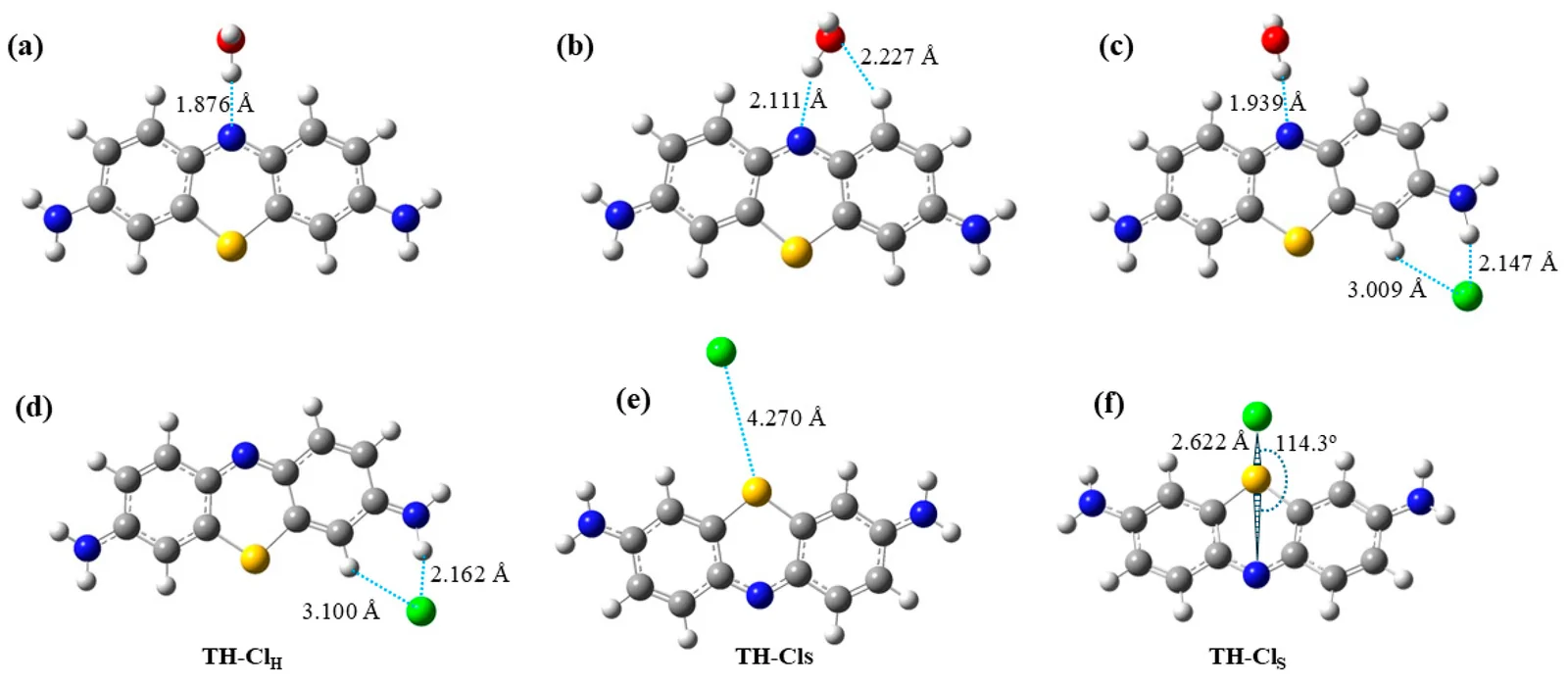
Calculated minimum structures of (**a**) TH-W (gas phase), (**b**) TH^+^-W (gas phase), (**c**) TH-Cl-W (in water solvent), (**d**) TH-Cl_H_ (gas phase), (**e**) TH-Cl_S_ at B3LYP/def2-TZVP (in water solvent) and (**f**) TH-Cl_S_ at B3LYP/def2-TZVP (in gas phase). atoms: grey balls, H atoms: white, O atoms: red; N atoms: blue, S atoms: yellow, Cl atoms: green.

**Figure 3:**
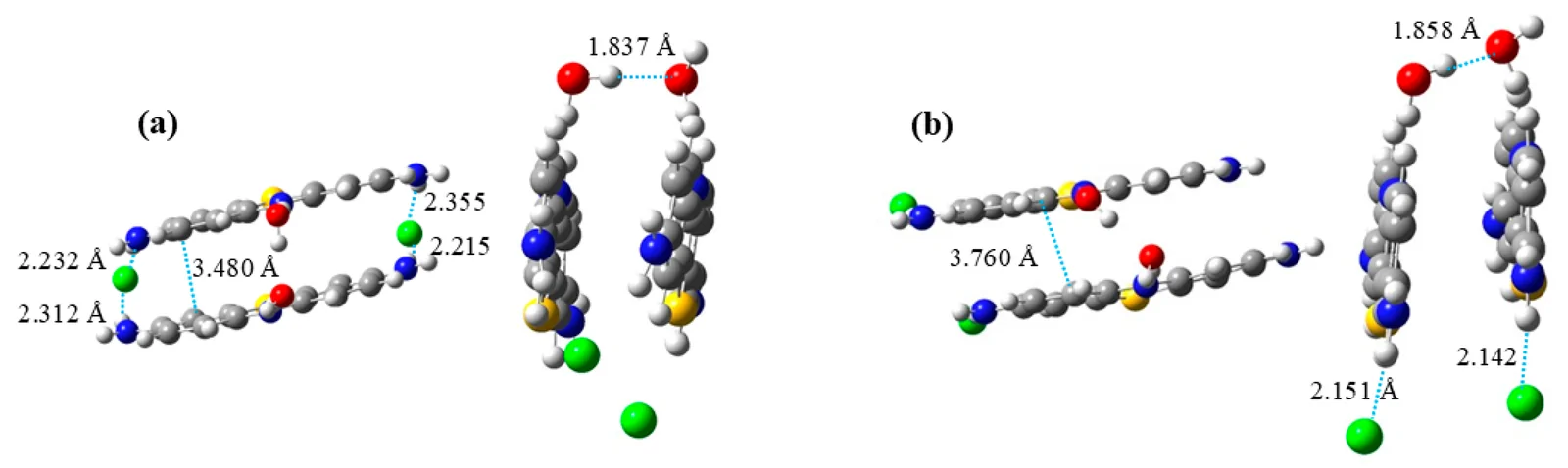
Calculated minimum structure of (**a**) (TH-Cl-W)_2_-1 and (**b**) (TH-Cl-W)_2_-2 at ωB97XD/6-31G (d, p) in water solvent.

Geometry: The calculated minimum structures of TH-W, TH^+^-W, TH-Cl_H_, TH-Cl_S_, TH-Cl-W, and (TH-Cl-W)_2_ minimum structures in water solvent are given in Figure 2 and Figure 3. A water molecule of the solvent has been added explicitly; it forms an OH^…^N hydrogen bond with TH or TH^+^ species that ranges from 1.88 to 2.11 Å. In the case of the TH^+^-W, the water molecule forms an additional van der Waals bond, and a six-member ring is formed; see Figure 2b.

In solid, thionine is an organic chloride salt composed of 3,7-diaminophenothiazin-5-ium and chloride ions in a 1:1 ratio, and the Cl^−^ anion interacts with the S atom of the TH^+^. Different structures where the Cl^-^ interacts with H of -NH_2_ or of a -CH group, with N, and with S were investigated. All geometry optimizations resulted in two minimum structures, where Cl^-^ interacts with the H atom of -NH_2_, i.e., TH-Cl_H_, or Cl^-^ interacts with S, i.e., TH-Cl_S_; see Figure 2. It is found that, in solution, Cl^-^ prefers to form a noncovalent interaction with the H of the -NH_2_ group, while the interaction of Cl^-^ with the S is a local minimum structure, lying 0.34 eV above the global minimum structure at the B3LYP/def2-TZVP method. Note that the S^…^Cl distance in the gas phase is significantly shorter by 1.6 Å than in water solvent when the solvent is added only implicitly. For this reason, additional calculations have been carried out where the first solvation sphere has been added explicitly; see the discussion below.

Furthermore, the aggregation of the TH^+^ in the presence of Cl^-^ has been studied in water solvent, while two molecules of the solvent have been added explicitly; see Figure 3. Thus, dimers of the TH-Cl-W species have been studied. Different structures of (TH-Cl-W)_2_ were investigated, including also structures where the TH-Cl-W molecules are perpendicular; see Figure S1 in Supplementary Materials. Geometry optimization resulted in the parallel structures depicted in Figure 3. Two stable dimers, (TH-Cl-W)_2_-1 and (TH-Cl-W)_2_-2, have been obtained, which are stabilized via π-stacking interactions; see also the discussion below regarding the molecular orbitals, i.e., π-π distances calculated at 3.480 Å and 3.760 Å, typical π-π interactions [56]. Additionally, the two water molecules that form N^…^HOH bonds with the TH^+^ cations also form a hydrogen bond between them, i.e., HOH^…^OH_2_ with a bond distance of about 1.85 Å; see Figure 3. The (TH-Cl-W)_2_-1 and (TH-Cl-W)_2_-2 structures differ in the relevant positions of the Cl^-^ anions, i.e., at (TH-Cl-W)_2_-1, the two Cl^-^ interact with -NH_2_ groups of the two TH^+^ that are not adjacent, while at (TH-Cl-W)_2_-2, the two Cl^-^ interact with -NH_2_ groups of the two TH^+^ that are neighboring; see Figure 3. As a result, at the (TH-Cl-W)_2_-1 dimer, each Cl^-^ interacts with the -NH_2_ groups of both TH^+^, and thus four Cl^…^HN bonds are formed, with their distances ranging from 2.215 Å to 2.355 Å. Thus, (TH-Cl-W)_2_-1 is more stable than (TH-Cl-W)_2_-2 by 1.88 eV at ωB97XD/6-31G(d,p), where at (TH-Cl-W)_2_-2 two Cl^…^HN bonds are formed with bond distances of about 2.15 Å; see Figure 3. The Gibbs formation energies of the dimers in water solvent at ωB97XD/6-31G(d,p) are calculated at ΔG = −9.11 kcal/mol for (TH-Cl-W)_2_-1 and ΔG = −4.11 kcal/mol for (TH-Cl-W)_2_-2, confirming that dimerization is energetically favorable.

**Figure 4:**
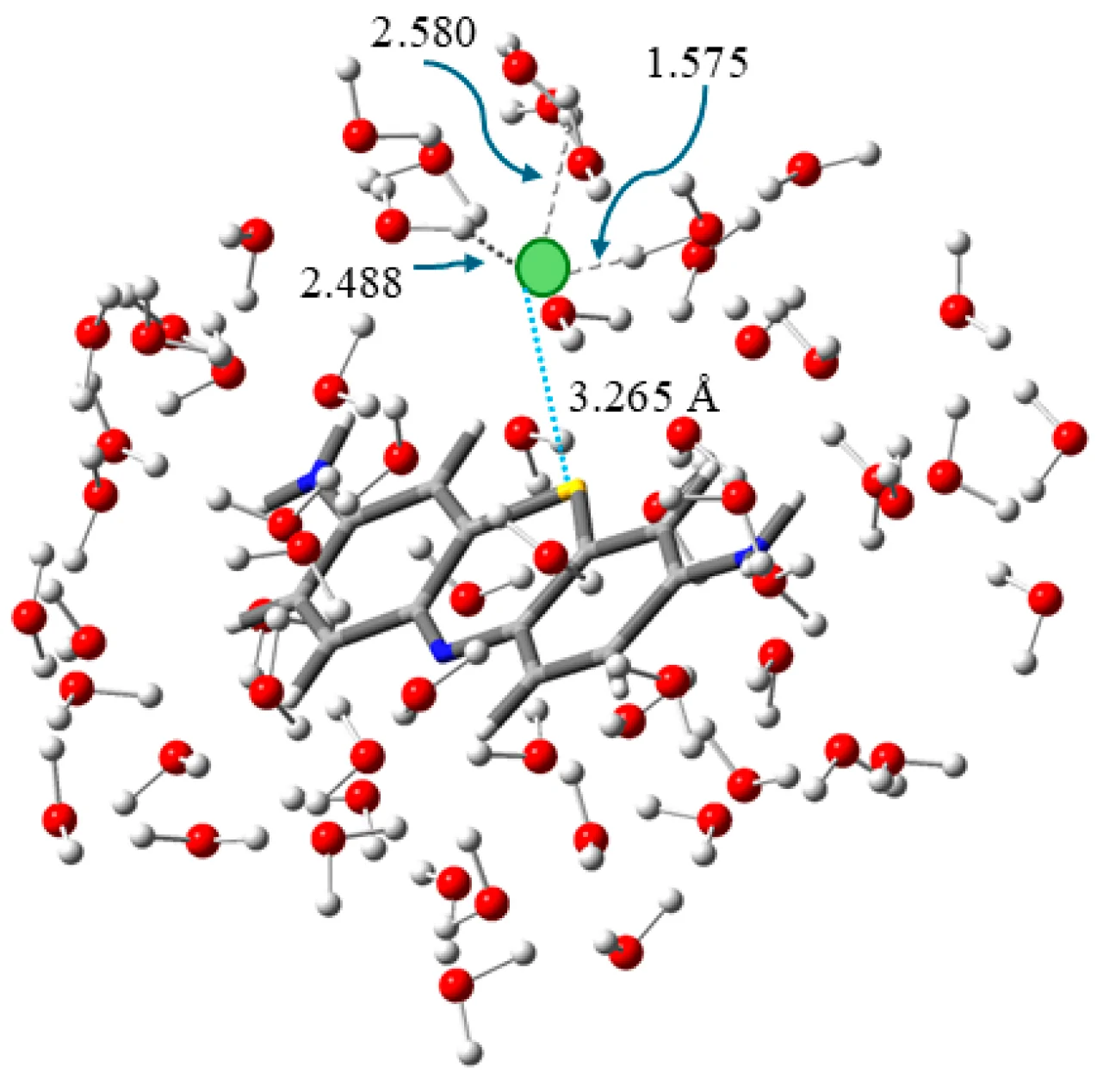
Calculated minimum structure of TH-Cl_S_@W61 in ONIOM(DFT:PM6) methodology.

Finally, as shown in Figure 3, the Cl^-^ anion prefers to be attached at -NH_2_, while when it is attached to the S of the central ring, the minimum is a local minimum structure. So, instead of adding the solvent via dielectric constant, 61 water molecules have been added around the TH-Cl, which correspond to the first sphere of solvation; see Figure 4. Initially, we had a model with 100 water molecules that was optimized at the PM6 level of theory. Then, the model was reduced to 61 water molecules for the ONIOM calculation. The TH-Cl has been calculated at the DFT level of theory and water molecules at the PM6 level of theory. This number was chosen to encompass all water molecules located within 2.6 Å of any atom of thionine or of the Cl^−^ counterion while maintaining an approximately balanced number of water molecules above and below the plane of the thionine.

The geometry of the system was energetically optimized at the ONIOM(B3LYP/6-31G(d,p):PM6) methodology. The size of the model is 13.4 × 13.7 × 15.9 Å. Note that, for the TH-Cl_S_ species, in the gas phase, the S^…^Cl distance is calculated at 2.622 Å, while in aqueous solution, when the solvent is added only explicitly, the S^…^Cl distance is increased at 4.353 Å. However, when 61 water molecules are added explicitly, the S^…^Cl distance is reduced at 3.265 Å, i.e., the existence of the water molecules stabilizes the S^…^Cl bond, and the S^…^Cl distance is reduced by 1 Å, showing the importance of the explicit inclusion of water molecules. Furthermore, Cl^−^ forms three additional hydrogen bonds with water molecules that range from 1.57 to 2.58 Å. The TH-Cl@W_61_ structure was calculated to examine the effect of the addition of the first sphere of solvation instead of one water molecule—see Figure 2—on the absorption spectra—see the discussion below. Overall, the inclusion of the explicit water molecules further stabilizes the Cl^−…^ S^+^ interaction. It should be noted that even though the TH-Cl_S_@W61 system has been optimized, this structure represents one microsolvated configuration, rather than an ensemble-averaged description of aqueous solvation.

**Figure 5:**
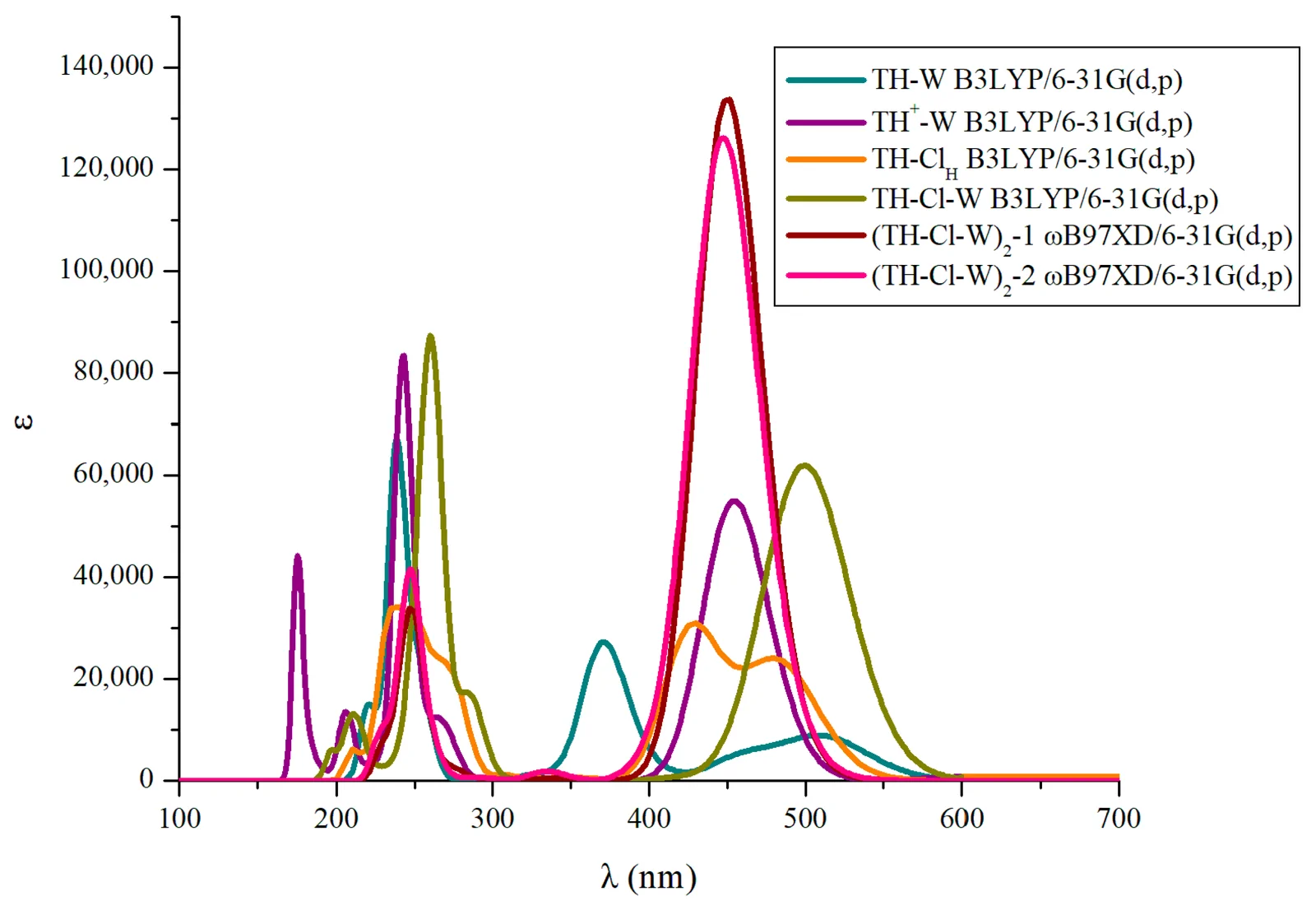
Absorption spectra of the calculated minimum structures of thionine in water solvent. (UV-vis peak half-width at half-height is 0.22 eV).

Absorption Spectra: Selected absorption λ and oscillator strength (f) values of the calculated minimum structures of TH-W, TH^+^-W, TH-Cl_H_, TH-Cl_S_, TH-Cl-W, and (TH-Cl-W)_2_ in various levels of theory in gas phase, water solvent and benzene solvent are given in Table 1. Depending on the calculated species, TH-W, TH^+^-W, TH-Cl_H_, TH-Cl_S_, TH-Cl-W, and (TH-Cl-W)_2_ the first vis peak is observed in the region that ranges from 571 nm (TH^+^-Cl_s_) to 462 nm (TH^+^-Cl_H_) in water solvent; see Table 1 and Figure 5. The (TH-Cl-W)_2_-1 that corresponds to the aggregation structures presents a weak peak, which is a charge transfer excitation at 634 nm and an intense peak at 481 nm. Experimentally, Nicotra et al. [16] presented an absorption spectrum of thionine in aqueous solution at different pH values. A clear absorption peak was obtained at 599 nm in pH = 5.5–10.7, while the main peak is at 510 nm in pH = 11.6–12.3. These peaks have been attributed to the presence of the thionine monomer with a positive resonant charge [16]. The present main calculated vis absorption peaks were found to be at 495 nm (TH-Cl_H_) and 503 nm (TH-Cl-W) in water solvent. In the gas phase, the lowest in energy peak in the vis region is weak, while the second one is the most intense; however, in water solvent, the opposite occurs; see Table 1. Finally, the effect of the cLR approach on the calculation of the main peaks is not important, i.e., a small shift of 14 nm was observed.

Overall, all the TH-W, TH^+^-W, TH-Cl_H_, TH-Cl_S_, TH-Cl-W, and (TH-Cl-W)_2_ minimum structures have some intense peaks in the visible region between 400 and 600 nm as well as in the ultraviolet area between 150 and 360 nm. Concerning the absorption spectra of the dimers, an intense peak can be observed at almost the same wavelength, i.e., 450.7 nm for (TH-Cl-W)_2_-1 (dark red line) and 446.2 nm for (TH-Cl-W)_2_-2 (pink line), as shown in Figure 5. Each dimer also has a smaller peak at 524.4 nm (dimer 1) and at 521.0 nm (dimer 2), but the oscillator strength is very small (f_1_ = 0.0012, f_2_ = 0.0013) for both peaks. Note that thionine in all TH^+^-W, TH-Cl_H_, TH-Cl_S_, TH-Cl-W, (TH-Cl-W)_2_ and TH-Cl_S_@W_61_ is positively charged and Cl^-^ is negatively charged and thus all molecular systems are neutral. TH-W is a neutral molecular system, i.e., open-shell system, and the thionine is neutral. Thus, TH-W and TH^+^-W, where there is no Cl^-^ anion, are open- and closed-shell systems, respectively. We observe that the neutral TH has less intense main absorption peaks than the positively charged TH^+^; see Figure 5.

**Figure 6:**
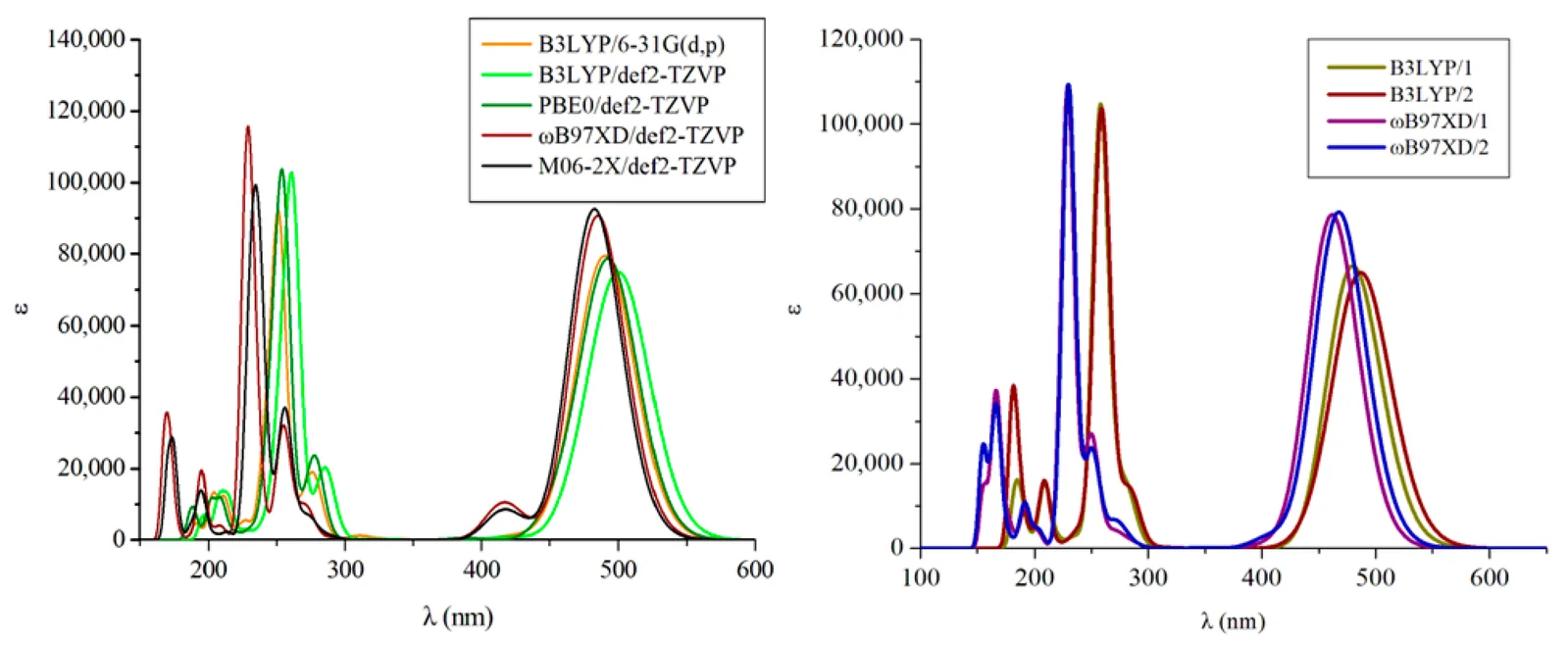
Left: Absorption spectra of the calculated minimum structures of TH-Cl-W in different methodologies in water solvent. Right: Absorption spectra of the calculated intermediate 1: TH-Cl_H_ and 2: TH-Cl_S_ in B3LYP/def2-TZVP and ωB97XD/def2-TZVP in water solvent. (UV-vis peak half-width at half-height is 0.22 eV).

The effect of different functionals in conjunction with different basis sets on possible shifts in the spectra has been investigated. The results indicated negligible shifts in the spectra; see Table 1 and Figure 6. In particular, the first intense peak of each structure is observed at about 480–500 nm. More precisely, at the B3LYP/6-31G (d, p) spectrum (dark yellow line), a peak is obtained at 493.1 nm. The B3LYP/def2-TZVP spectrum (light green line) exhibits a peak at 503.3 nm, while the PBE0/def2-TZVP spectrum (dark green line) presents a peak at 494.3 nm. Similarly, the ωB97XD/def2-TZVP (dark red line) and M06-2X/def2-TZVP (black line) spectra present peaks at 485.3 nm and 482.8 nm, respectively; see Figure 6.

**Figure 7:**
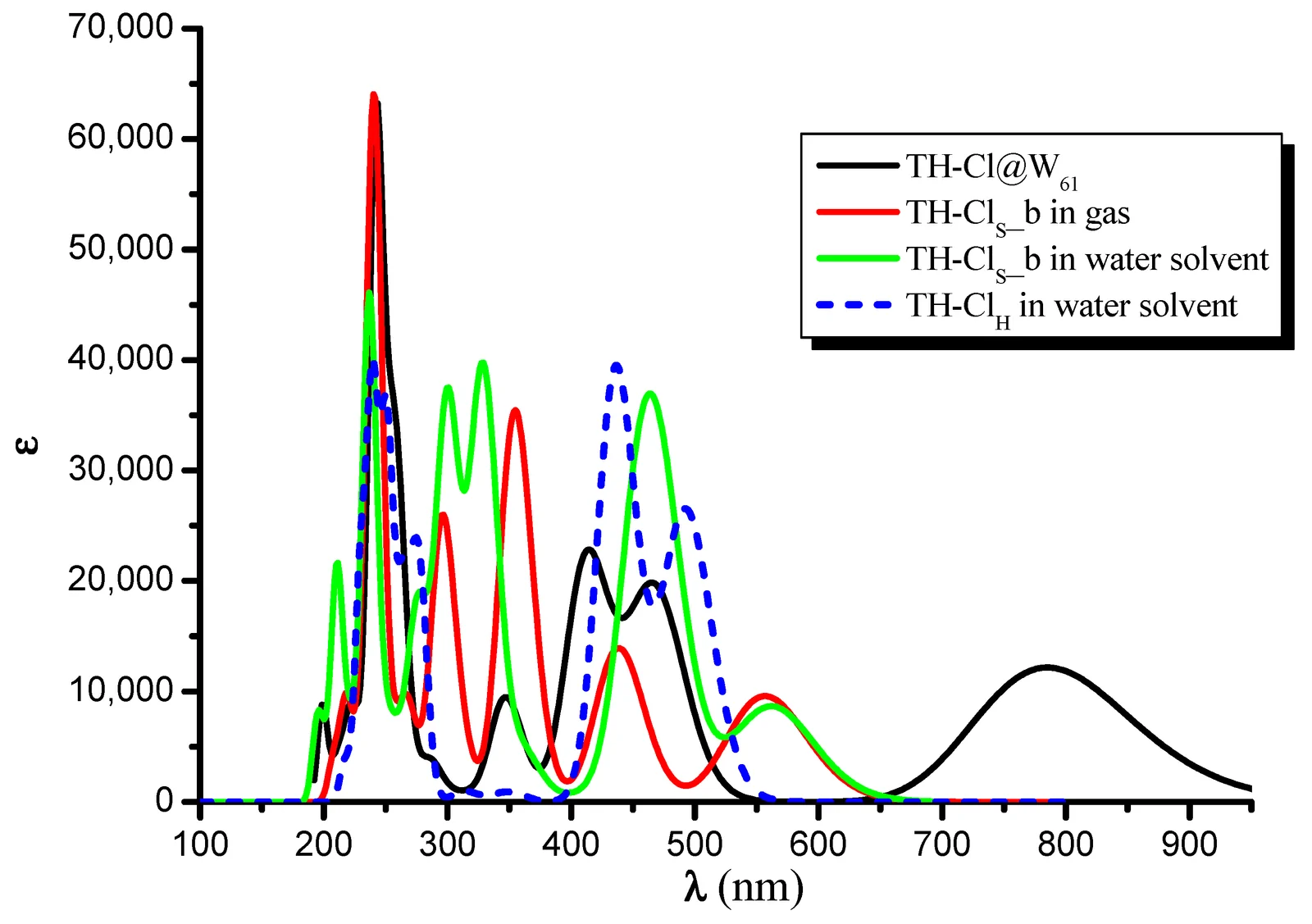
Absorption spectra of the calculated minimum structures of TH-Cl in the gas phase and in water solvent at B3LYP/def2-TZVP (UV-vis peak half-width at half-height is 0.22 eV).

The TH-Cl_S_ structure calculated in the gas phase, with a Cl^…^S distance at 2.622 Å, presents a peak at 571 nm. When the water solvent is added implicitly in this structure, the first peak is calculated at 563 nm, while the intensity of the second absorption peak at 464 nm is significantly increased in the solvent. However, when Cl is attached on the -NH_2_ group or the S^…^Cl distance is increased, the peak around 570 nm is blue-shifted, and the calculated absorption spectra are the same. Furthermore, to investigate the explicit inclusion of the solvent, additional calculations regarding TH-Cl_S_@W_61_ were carried out in different levels of theory, and all predict similar spectra. The calculated absorption spectra predict an additional peak at near-IR ~820 nm and intense peaks at ~480 nm and 460 nm; see Figure 7. Given that the vis absorption spectrum of thionine in aqueous solution at different pH values is expected in the region 510–600 nm [16,20] and our calculation shows peaks either in 570 nm or in 503 nm, the vibronic spectra will be calculated.

Finally, note that the ωB97XD functional includes an empirical dispersion contribution, and thus it is appropriate for the calculation of the dimers where noncovalent interactions are observed. However, the present study and the literature show that ωB97XD predicts blue-shifted absorption and emission spectra with respect to the experimentally measured spectra; see Table 1. Hence, B3LYP electronic spectra are in better agreement with the experimental ones, and this is the reason why the B3LYP functional is used for the calculation of the dimers’ spectra; see Figure 7.

**Figure 8:**
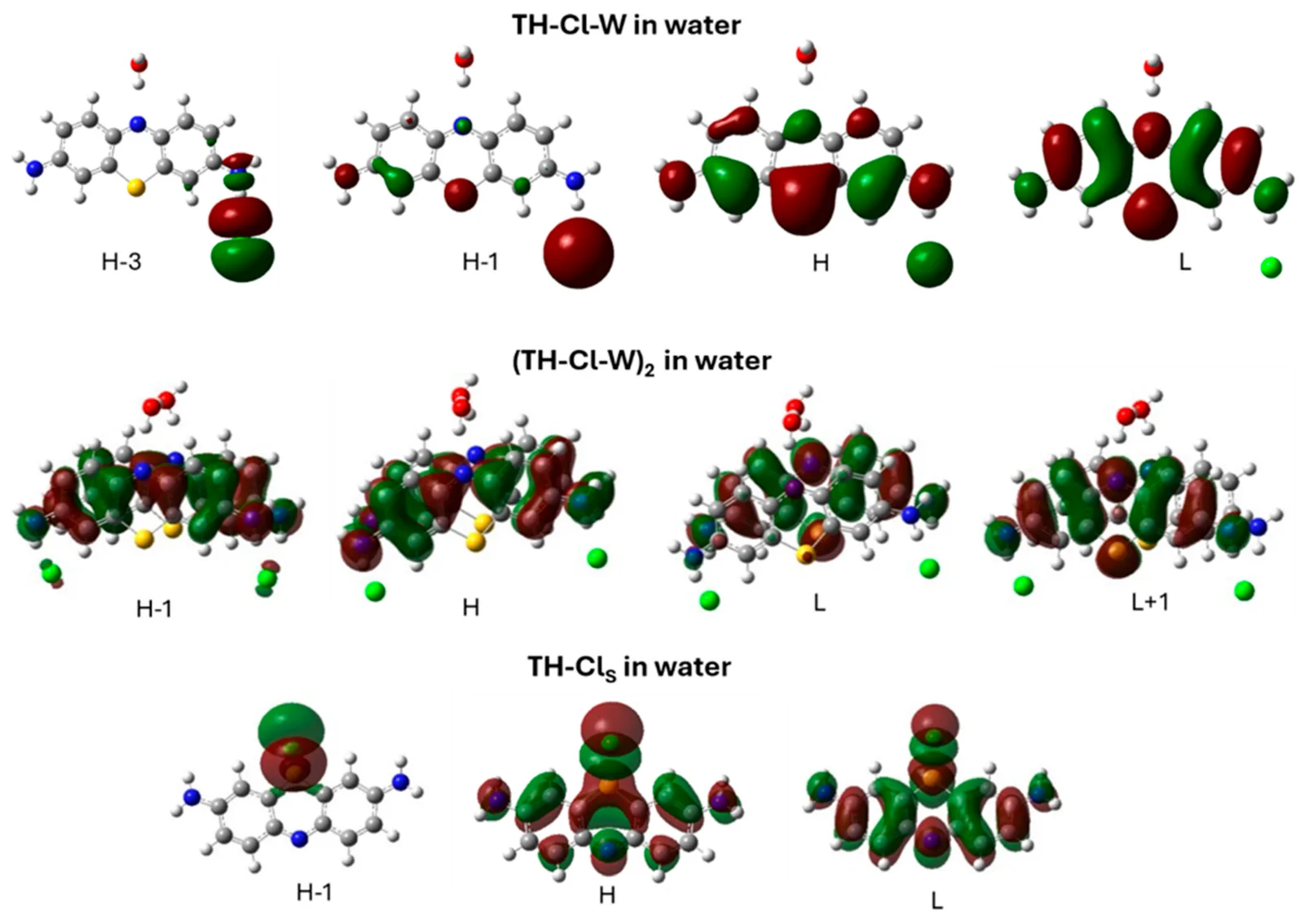
Frontier molecular orbitals of the TH-Cl-W, TH-Cl_S_ and (TH-Cl-W)_2_-1 at the B3LYP/def2-TZVP level of theory in water solvent.

Molecular Orbitals: For TH-Cl-W in water solvent, the main peak at 503 nm corresponds to a H→L excitation, while the absorption peaks at 478 nm and 420 nm correspond to H-1→L and H-3→L excitations, respectively, which are both charge transfer excitations from Cl to TH; see Figure 8. Similarly, for both TH-Cl_H_ and TH-Cl_S_ in water solvent, the second peak at ~467 nm is also a charge transfer excitation from Cl to TH. However, for TH-Cl_S_, when Cl^−^ is close to the S atom, i.e., the S^…^Cl distance is ~2.6 Å, H-1 and H are reversed, and the first peak is observed around 570 nm; its intensity is reduced and corresponds to a charge transfer excitation from Cl to TH.

For the (TH-Cl-W)_2_ dimers, H-1 and H are almost energetically degenerate, while the same applies to the L and L+1 orbitals. Their electron density is localized in the whole dimer, while it is observed electron density between the two TH monomers. For the (TH-Cl-W)_2_-1 dimer, a weak peak at ~600 nm is observed, which corresponds to the H→L excitation, while the absorption peak at 481 nm corresponds to a H-1→L excitation; see Figure 8. Finally, Natural Transition Orbitals (NTOs) have been calculated (see Figure S3), showing only negligible differences with the molecular orbitals. This occurs because the coefficient of the main excitation is larger than 0.9, i.e., a nearly pure one-electron transition.

### 3.2. Methylene Blue

**Figure 9:**
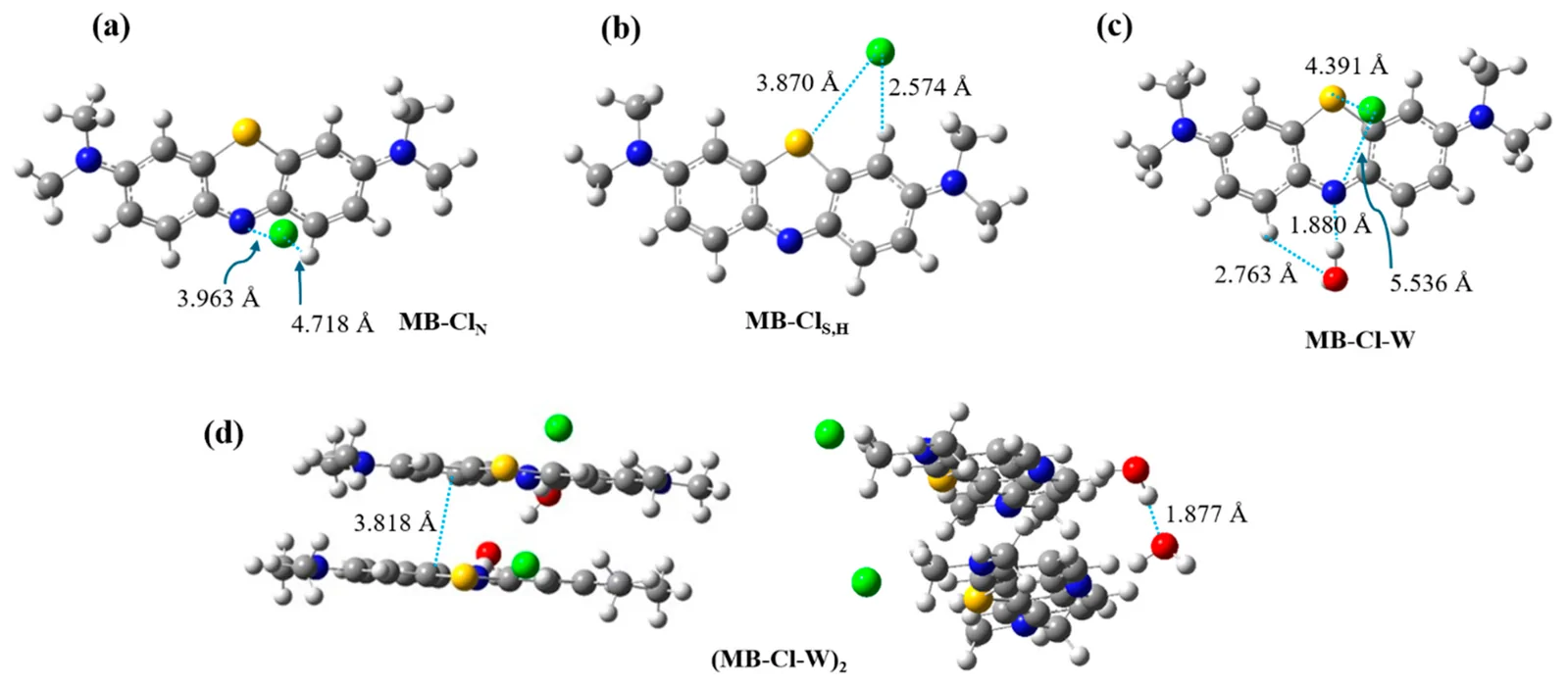
Calculated minimum structures of (**a**) MB-Cl_N_, (**b**) MB-Cl_S,H_, (**c**) MB-Cl-W at B3LYP/def2-TZVP, and (**d**) (MB-Cl-W)_2_ via two different points of view at the ωB97XD/def2-TZVP level of theory in water solvent.

Geometry: The calculated minimum structures of MB-Cl_N_, MB-Cl_S,H_, MB-Cl-W, and (MB-Cl-W)_2_ are shown in Figure 9. Given that MB has tertiary amine groups instead of primary amine groups, different types of minima structures are observed compared to the TH. Thus, the Cl^-^ anion interacts with either the S or N atoms of the central ring. In MB-Cl_S,H_, Cl^-^ forms both the H^…^Cl noncovalent interaction of 2.574 Å and S^…^Cl of 3.870 Å. In MB-Cl_N_, Cl^-^ interacts with N through an elongated N···Cl noncovalent interaction of 3.963 Å. A water molecule of the solvent has been added explicitly; it forms an OH^…^N hydrogen bond MB species of about 1.880 Å. The dimerization of the MB in the presence of Cl^-^ has been studied in water solvent, while two molecules of the solvent have been added explicitly; see Figure 9. The (MB-Cl-W)_2_ dimer is stabilized via π-stacking interactions, i.e., the π-π distance is 3.818 Å, and the formation of a hydrogen bond; see Figure 9d. Additionally, the two water molecules that form N^…^HOH bonds with the MB^+^ cations form also a hydrogen bond between them, i.e., HHH^…^OH_2_ of 1.877 Å.

**Figure 10:**
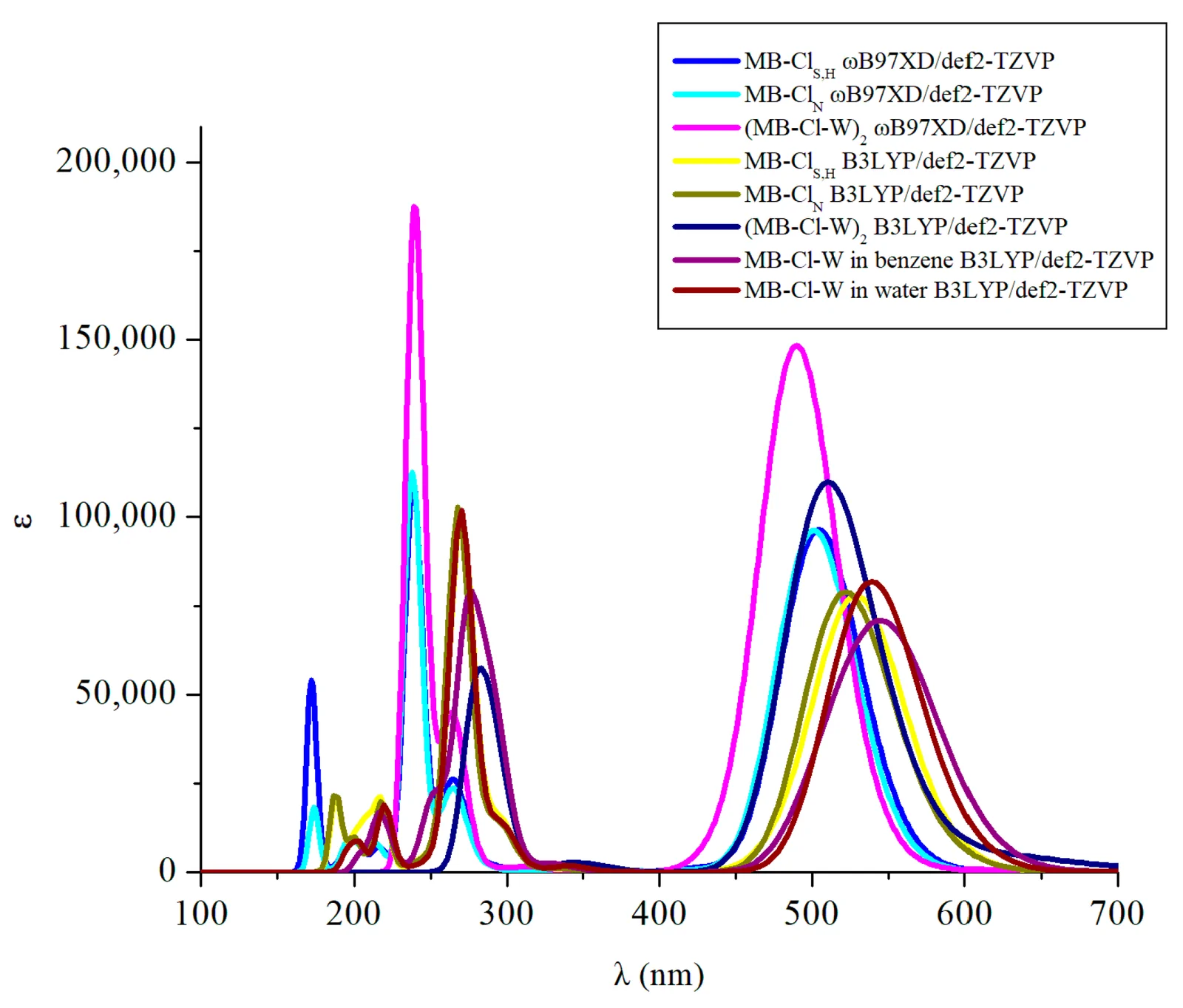
Absorption spectra of calculated minimum structures of methylene blue in water solvent. (UV-vis peak half-width at half-height is 0.22 eV).

Absorption spectra: Experimentally, Palit and Saxena [20] measured the absorption spectra of methylene blue in water, buffer pH 11.2, benzene, water, and dry benzene. The main peak of the absorption spectra is shifted, depending on the solvent, i.e., the main peak is observed in the region 500–670 nm. In the cases where methylene blue is in benzene and dry benzene, the main peaks are around 510–530 nm, while in water, the peak is observed in the region 610–665 nm. In the present study, the main vis peak depending on the molecular structure is observed in the region 555–491 nm, while a weak peak is observed in the region 570–700 nm; see Table 2 and Figure 10. Overall, there is fair agreement with the experimental values, with observed shifts of about 30 nm; however, the study of the vibronic spectra will improve the agreement.

Finally, the calculated MB molecular structures present strong absorption peaks in the region 220–300 nm. MB-Cl-W presents an absorption peak at 290 nm, in very good agreement with the experimental value of 293 nm [18,57].

**Figure 11:**
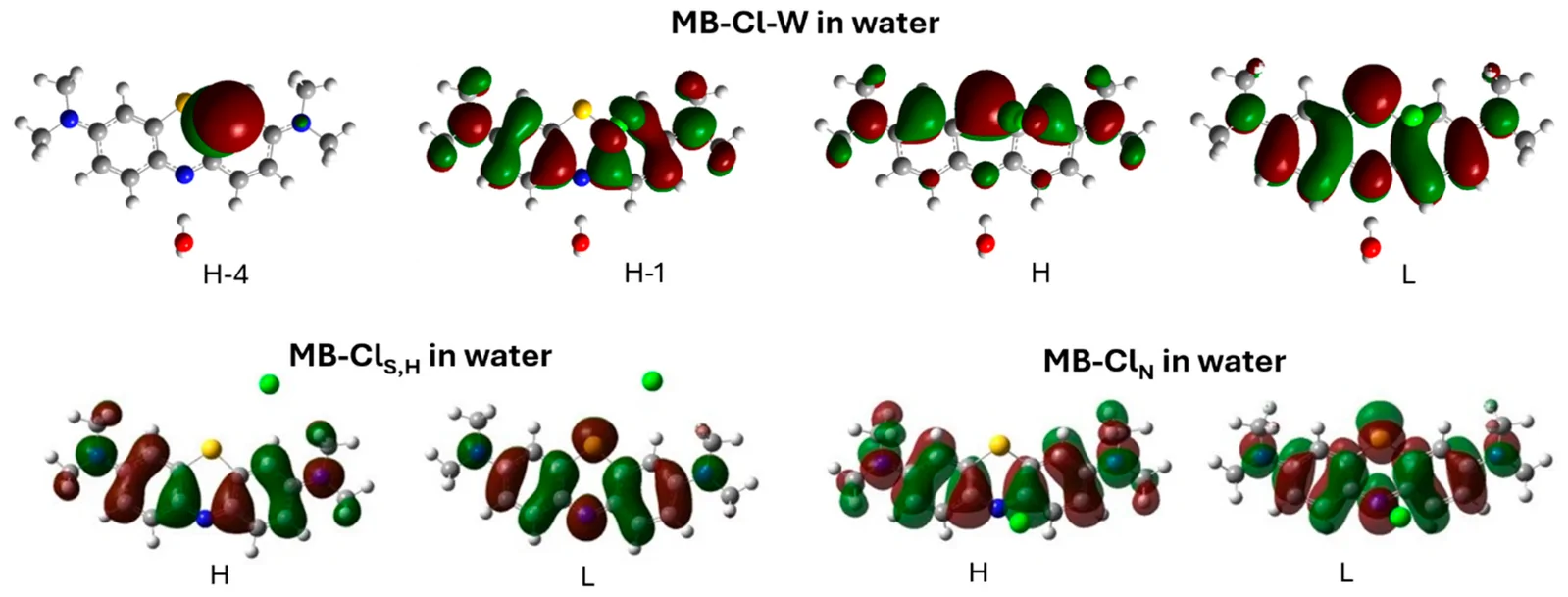
Frontiers molecular orbitals of MB-Cl-W, MB-Cl_N_ and MB-Cl_S,H_ at the B3LYP/def2-TZVP level of theory in water solvent.

Molecular Orbitals: The first absorption peak at 530 nm for MB-Cl_S,H_, 555 nm for MB-Cl_N_ and 565 nm for MB-Cl-W correspond to a H→L excitation. A small charge transfer is observed within the molecular system; for instance, there is no electron density in the S atom in the case of the H orbital of both MB-Cl_S,H_ and MB-Cl_N_—see Figure 11—while in L, electron density is transferred to the S atom. Electron transfer excitations are observed in the case of MB-Cl-W; for instance, the peak at 501 nm is an electron transfer excitation from Cl^-^ to the whole MB and corresponds to a H-4→L excitation; see Figure 4. Finally, for (MB-Cl-W)_2_, the peak at 700 nm corresponds to a H→L excitation; however, in both H and L orbitals, the electron density is localized in both MB molecules. Note that the calculated molecular orbitals show only negligible differences upon NTO application; compare Figure 11 with Figure S3 of Supplementary Materials.

### 3.3. Emission Spectra

The S_1_→S_0_ and S_2_→S_0_ de-excitations of the calculated structures of both methylene blue and thionine with a Cl^-^ anion attached close to the S atom have been calculated at the B3LYP/def2-TZVP level of theory in water solvent; cLR was also employed to ensure that the values obtained are the actual ones; see Table 3. The molecular orbitals involved in the de-excitation are also given in Table 3. The S_1_→S_0_ of TH_s_-Cl is an electron transfer de-excitation from the TH^+^ species to the Cl^-^ anion. Note that the S_2_→S_0_ of TH_s_-Cl and both S_1_→S_0_ and S_2_→S_0_ of MB-Cl_S,H_ also correspond to a charge transfer de-excitation from the TH^+^ species to the Cl^-^ anion, but both TH^+^ and MB^+^ retain electron density. The S_1_→S_0_ peak was calculated at 666(657) nm and 642 nm for MB_S,H_-Cl and TH_s_-Cl, respectively. Experimentally, MB and TH in water solvent present emit at 686 [18] and 620 nm [58]. There is a good agreement with the experimental values.

### 3.4. Vibronic Spectra

**Figure 12:**
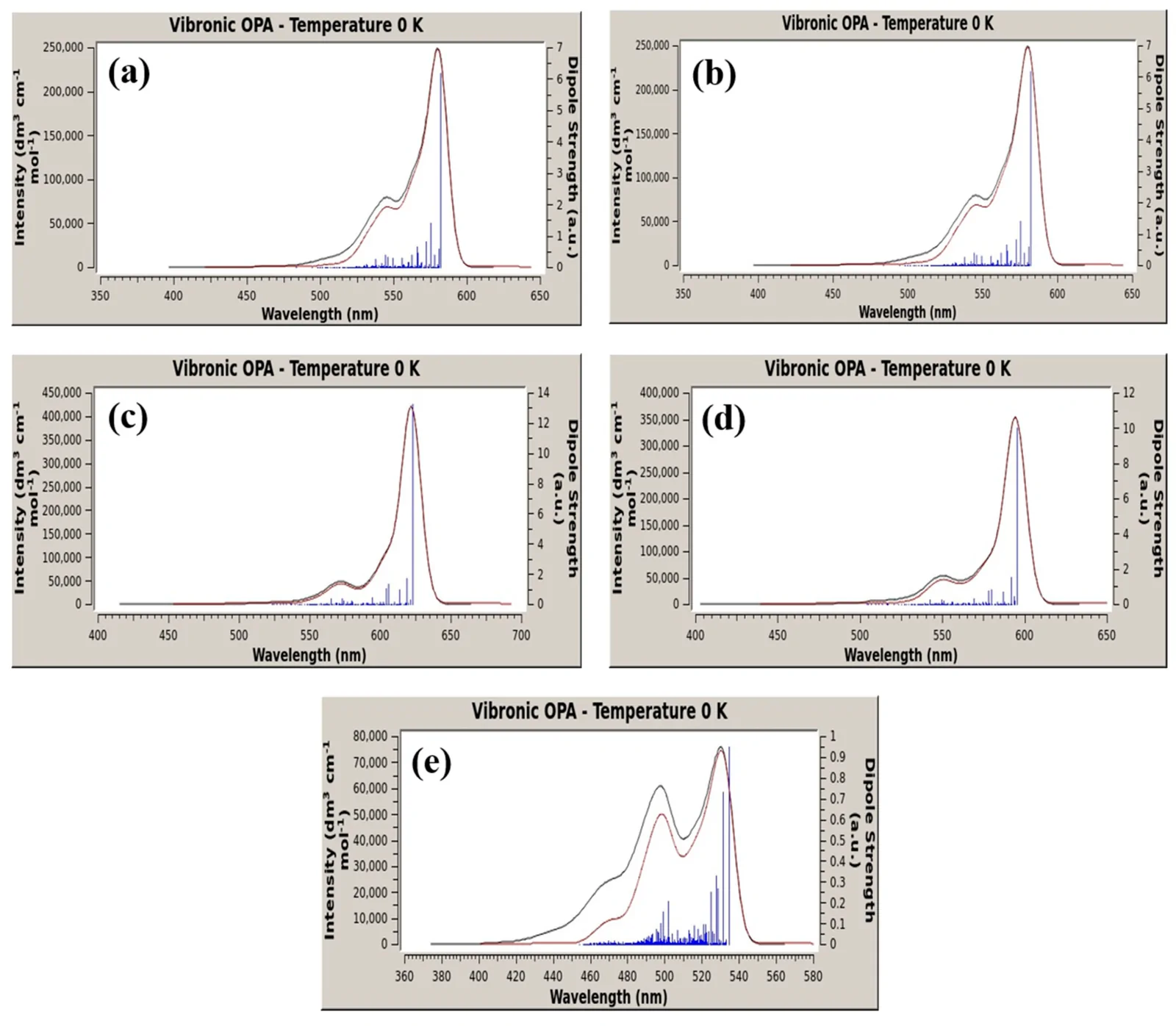
Vibronic absorption spectra of (**a**) TH-Cl-W in water solvent, (**b**) TH^+^ in water, (**c**) MB^+^ in water, (**d**) MB^+^ in chloroform, and (**e**) MB-S^+^ in gas phase at B3LYP/6-31G(d,p).

The absorption vibronic spectra of TH-Cl-W, TH^+^, and MB^+^ at B3LYP/6-31G(d,p) water solvent and chloroform solvent and in the gas phase have been calculated; see Figure 12. Due to technical issues with the use of the def2-TZVP basis set for the calculation of the vibronic spectra, only the 6-31G(d,p) basis set was employed. In Figure 12, the *black line* shows the final spectrum (Gaussian Broadening, HWHM = 200 cm^−1^), the *blue vertical lines* show the reported transitions, right *Y*-axis, while the *red line* shows the spectrum from reported transitions (Gaussian Broadening, HWHM = 200 cm^−1^). The |0>→|0> vibronic transition was calculated at 582 nm for TH-Cl-W and 559 nm for TH^+^ in water solvent; see Table 4. These values are in excellent agreement with the experimental ones that range in the region from 560 nm to 599 nm in water solvent. For MB^+^, the |0>→|0> vibronic transition was calculated at 622 nm in water solvent, which is again in excellent agreement with the experimental ones that range in the region from 610 nm to 644 nm. In chloroform and in the gas phase, it was found that |0>→|0> is blue-shifted at 595 nm and 534 nm, respectively; see Table 4. Thus, the inclusion of vibrational effects improves the calculated spectra in the visible region, whereas for the calculation of peaks in the UV region, vibronic spectra are not required.

## 4. Comparison Between Thionine and Methylene Blue

Thionine and methylene blue are two well-known photosensitizers with favorable applications. It is known that both can generate singlet oxygen. More specifically, thionine is useful particularly in histology as it exhibits high affinity for cellular membranes. It also shows selectivity for tumor and microbial cells, which appears to be based on electrostatic interactions between the positive charge of these molecules and the negative charge found on the outer membrane/envelope of the target cell. On the other hand, methylene blue is an inexpensive photosensitizer used for antimicrobial photodynamic therapy and as a potent PDT drug for local treatment of periodontal diseases due to its efficiency against a broad spectrum of microbes including bacteria, fungi, and viruses [18]. The scope of the present study is not to evaluate cell interaction, tissue penetration or the clinical use of MB and TH but to investigate if the different calculated molecular systems and specifically the dimers, which are the smallest aggregation systems, can affect their photophysical properties, including their potential as a sensitizer for PDT.

**Figure 13:**
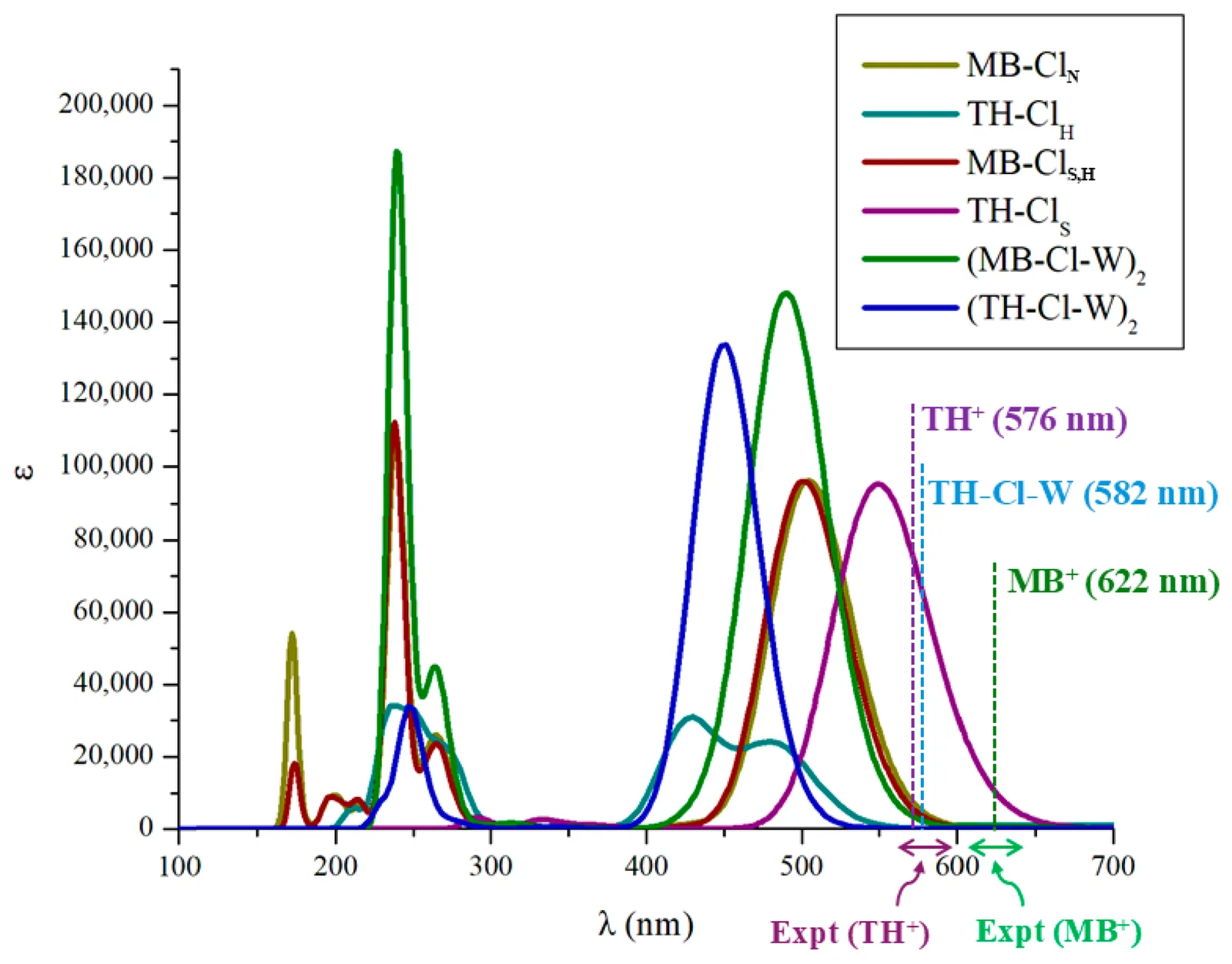
Absorption spectra of methylene blue and thionine minimum calculated structures, vibronic absorption peaks at the B3LYP/def2-TZVP level of theory in water solvent and experimental measured absorption peaks (Expt) (UV-vis peak half-width at half-height is 0.22 eV).

Comparing MB-Cl with TH-Cl, MB-Cl-W with TH-Cl-W, and (MB-Cl-W)_2_ with (TH-Cl-W)_2_, interesting observations are obtained. It seems that the oscillator strength values are much higher in methylene blue structures compared to the corresponding values of the thionine structures—see Figure 13—with the exception of the dimers, where the f-value is smaller for (MB-Cl-W)_2_ than for (TH-Cl-W)_2_. Overall, the calculated absorption spectra of the thionine structures exhibit lower intensities than those of the corresponding methylene blue structures, providing preliminary support for MB as a more promising photosensitizer candidate than TH. Note that stronger absorption supports better light harvesting, but photosensitizer performance also depends on triplet-state formation, triplet lifetime, singlet oxygen quantum yield, localization, and photostability.

Furthermore, the MB peaks are red-shifted compared to those of thionine by about 40 nm. The main vis peak of the dimers turned out to be blue-shifted by 22(37) nm with respect to TH-W-Cl(MB-Cl-W). Finally, incorporating vibrational effects enhances the accuracy of the computed spectra in the visible region, while vibronic spectra are unnecessary for reproducing the UV-region peaks. Note that the calculated vibronic peaks are ~80 nm red-shifted relative to the absorption peaks. The calculated absorption peaks are blue-shifted with respect to the experimental values, while the vibronic peaks are found in the region of the experimentally measured absorption peaks; see Table 5. Experimentally, Ganeev et al. [12] measured the absorption spectra of MB^+^ and TH^+^ in aqueous and ethanol solutions with the use of different concentrations. MB^+^ was found to absorb higher wavelength values than TH^+^, in agreement with our calculations. Also, it was experimentally observed that the dimers show blue-shifted bands compared to the bands of the monomers and appear at higher concentrations, in agreement with our calculations. Thus, the dimeric chromophores as minimal aggregated models can be used to “fine-tune” the photophysical properties.

It is known that a good photosensitizer generates ROS from its triplet state; the photosensitizer transfers energy or electrons to molecular oxygen. Note that the singlet oxygen, O_2_(^1^Δ_g_), lies approximately 0.98 eV above ground-state molecular oxygen O_2_(X^3^Σ^−^_g_). A good type II photosensitizer should have a T_1_ state lying about >0.98 [59] or >1.13 eV [60], as determined by experimental studies. For species whose T_1_ energies lie above the energetic requirement for ^1^O_2_ formation, the calculations indicate the energetic feasibility of singlet oxygen sensitization. The calculated T_1_ states of the molecular systems lie 0.86–1.44 eV above the S_0_ state; see Table 6 and Table S2. Only for TH-Cl_H_, a T_1_ state lying lower than the 0.98 eV was found. For the remaining molecular systems, the T_1_ state lies above this limit. The T_1_ states of (TH-Cl-W)_2_-1 and (MB-Cl-W)_2_ lie at 1.11 eV and 1.19 eV, respectively, very close to the energy demands of the ROS generation.

**Figure 14:**
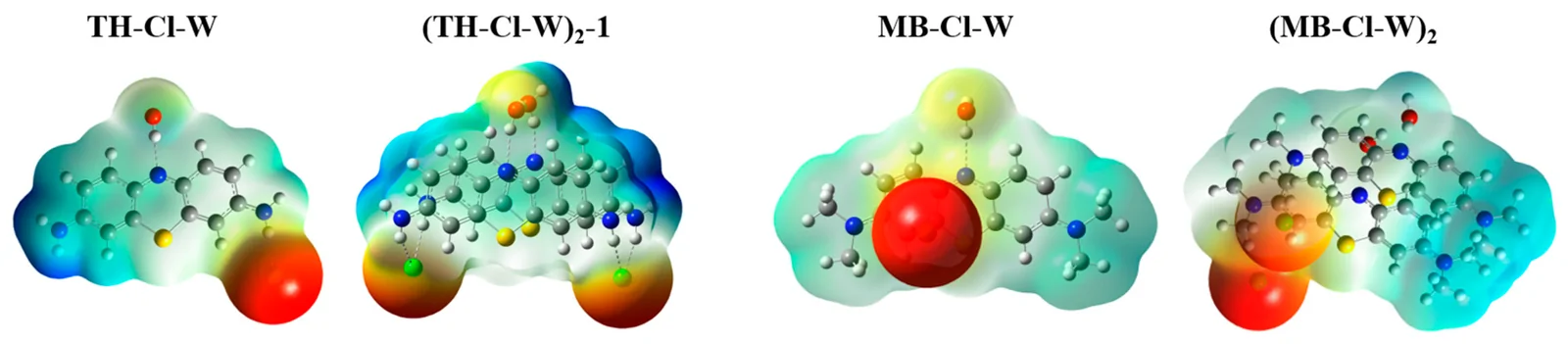
ESP of TH-Cl-W, MB-Cl-W, (TH-Cl-W)_2_ and (MB-Cl-W)*_2_* at B3LYP/def2-TZVP.

Finally, the electrostatic-potential (ESP) of the (MB-Cl-W)_2_ and (TH-Cl-W)_2_, together with those of the corresponding monomers MB-Cl-W and TH-Cl-W, are presented in Figure 14. A clearly electron-rich region, shown in red, is observed around the Cl atom, whereas the -NH_2_ group corresponds to an electron-poor region interacting with Cl. Overall, TH exhibits more pronounced electron-poor regions than MB, which is as expected because it possesses terminal -NH_2_ groups rather than -N(Me)_2_ groups.

## 5. Conclusions

Photodynamic therapy (PDT) is a relatively new therapy method that first appeared in the late part of the last century. It has already been used in dermatology, ophthalmology, and dentistry, and it is seen by many as a promising route toward highly effective cancer treatments, driving intense efforts to discover and optimize efficient photosensitizers. The present study examines the photophysical properties of two known photosensitizers to provide insight into their properties. Furthermore, the present study investigates the effect of electrostatic interactions, via the inclusion of Cl^-^ anions, on the calculation of the absorption and emission spectra.

In the present study, the molecular structures and the photophysical properties of TH and MB cations, salts of them with Cl^-^ anions (TH-Cl and MB-Cl), salts including water molecules (TH-Cl-W and MB-Cl-W), and their homodimers [(TH-Cl-W)_2_ and (MB-Cl-W)_2_] have been studied theoretically, employing DFT in water and benzene solvents. Furthermore, the TH-Cl@W_61_ molecular system was calculated to examine the effect of the addition of the first sphere of solvation on the photophysical properties of TH-Cl. As far as we know, these molecular systems, i.e., salts and dimers, have not been theoretically studied before. Our results are summarized below:

All the calculated systems are stable minimum structures. MB in all the different types of molecular systems presents absorption peaks that are red-shifted by ~40 nm relative to TH, while the main visible dimer peak is blue-shifted by 22–37 nm with respect to the corresponding monomers (TH-Cl-W and MB-Cl-W).

All methylene blue (MB) derivatives generally exhibit substantially larger oscillator strengths than the corresponding thionine (TH) derivatives, leading to more intense absorption bands, apart from (MB-Cl-W)_2_ which shows a smaller f-value than (TH-Cl-W)_2_.

The computed spectra are significantly improved in the visible region upon the inclusion of vibrational effects in the absorption spectra. Their inclusion is not required for the UV region. Vibronic peaks are ~80 nm red-shifted relative to the purely electronic absorption peaks. The calculated electronic absorption peaks are blue-shifted compared to the experiment, whereas the vibronic peaks align closely with the experimentally measured absorption maxima, showing the importance of the inclusion of vibrational corrections. These trends agree with the experimental data [12] where MB^+^ absorbs at longer wavelengths than TH^+^ in aqueous and ethanol solutions, and dimers appear at shorter wavelengths than monomers and become more prominent at higher concentrations.

Finally, linkage of monomers into dimers has been found to lead to fine-tuning of their photophysical properties, while dimeric chromophores, which are the smallest aggregated structures, can be used in fields including photodynamic therapy, photocatalysis and photon upconversion.

## Figures and Tables

**Table 1:** Selected absorption λ values (nm) and oscillator strength f-values of the calculated minimum structures of TH-W, TH^+^-W, TH-Cl_H_, TH-Cl_S_, TH-Cl-W, (TH-Cl-W)_2_ and TH-Cl_S_@W_61_ in various levels of theory in the gas phase, in water solvent and in benzene solvent.

Molecule	Methodology	λ_1_	f	λ_2_	f	λ_3_	f
TH-W ^a^	B3LYP/6-31G(d,p)	513.0	0.0915	460.9	0.0540	396.4	0.0266
TH^+^-W ^a^	B3LYP/6-31G(d,p)	463.5	0.0097	454.5	0.6029	423.9	0.0005
TH-Cl_H_^a^	B3LYP/6-31G(d,p)	483.0	0.2558	427.5	0.3354	404.8	0.0006
^a^	B3LYP/def2-TZVP	525.9	0.0008	480.1	0.7322	469.1	0.0107
^b^	B3LYP/def2-TZVP	494.6	0.6510	466.7	0.1096	407.0	0.0063
^b^	ωB97XD/def2-TZVP	461.7	0.8758	413.1	0.0171	349.3	0.0014
TH-Cl_S_ ^a^	B3LYP/def2-TZVP	570.9	0.0156	555.1	0.0172	554.6	0.0754
^b,c^	B3LYP/def2-TZVP	562.5	0.0959	463.8	0.4109	328.6	0.4130
^b^	B3LYP/def2-TZVP	488.9	0.6729	465.6	0.0678	450.4	0.0328
^b^	ωB97XD/def2-TZVP	467.7	0.8828	409.7	0.0330	346.5	0.0014
TH-Cl-W ^b^	B3LYP/6-31G(d,p)	493.1 (478.6) ^d^	0.6300	471.1	0.1403	427.5	0.0120
^b^	B3LYP/def2-TZVP	503.3	0.5981	477.7	0.1451	420.8	0.0078
TH-Cl-W ^b^	B3LYP/def2-TZVP	497.4	0.6010	476.8	0.0829	466.5	0.0722
^b^	PBE0/def2-TZVP	494.3	0.6720	462.7	0.1122	387.7	0.0049
^b^	ωB97XD/def2-TZVP	485.3	0.8078	416.8	0.0939	327.0	0.0011
^b^	M06-2X/def2-TZVP	482.8	0.8239	417.2	0.0763	337.3	0.0010
^e^	B3LYP/def2-TZVP	746.7	0.0136	740.0	0.0000	499.5	0.5795
(TH-Cl-W)_2_-1 ^b^	B3LYP/def2-TZVP	634.3	0.0012	480.6	1.0669	420.3	0.0267
^b^	ωB97XD/6-31G(d,p)	524.4	0.0012	456.7	0.1647	450.7	1.0936
(TH-Cl-W)_2_-2 ^b^	ωB97XD/6-31G(d,p)	521.1	0.0013	466.4	0.2008	446.2	1.2314
TH-Cl_S_@W_61_	B3LYP/6-31G(d,p)	785.1	0.1353	468.9	0.1991	449.1	0.0223
TH-Cl_S_@W_61_	B3LYP/def2-TZVP	820.2	0.1171	477.8	0.1570	458.8	0.0135
TH-Cl_S_@W_61_	PBE0/def2-TZVP	786.8	0.1173	433.5	0.4467	412.9	0.0233
TH-Cl_S_@W_61_	ωB97XD/def2-TZVP	813.4	0.1206	464.5	0.1948	446.4	0.0197
TH-Cl_S_@W_61_	M06-2X/def2-TZVP	828.2	0.1114	434.1	0.4117	416.3	0.0310
Expt ^b,f^		560					
Expt ^b,g^		599 or 510		550			

^a^ In gas phase. ^b^ In water solvent. ^c^ Single-point calculation at gas phase geometry. ^d^ cLR correction. ^e^ In benzene solvent. ^f^ Reference [20] in water solvent. ^g^ Reference [16] in water solvent; 599 nm peak, 550 nm shoulder; peak at 510 nm for basic pH.

**Table 2:** Selected absorption λ values (nm) and oscillator strength f-values of the calculated minimum structures of MB-Cl_S,H_, MB-Cl_N_, MB-Cl-W, and (MB-Cl-W)_2_ in various levels of theory in water solvent and in benzene solvent have been calculated.

Molecule	Methodology ^a^	λ_1_	f	λ_2_	f	λ_3_	f
MB-Cl_S,H_^b^	B3LYP	530.3	0.8393	497.0	0.0195	488.1	0.0289
^b^	ωB97XD	504.5	1.0731	429.2	0.0134	353.2	0.0011
MB-Cl_N_ ^b^	B3LYP	554.5	0.0622	541.9	0.0478	520.0	0.8046
^b^	ωB97XD	501.3	1.0710	424.6	0.0060	367.2	0.0060
MB-Cl-W ^b^	B3LYP	565.0	0.0269	538.5	0.8805	500.8	0.0026
^c^	B3LYP	986.0	0.0085	554.4	0.6332	516.3	0.3011
(MB-Cl-W)_2_ ^b^	B3LYP	700.2	0.0041	514.6	0.3586	502.3	0.5375
^b^	ωB97XD	569.9	0.0057	491.4	1.5295	475.5	0.1286
Expt (MB) ^d^		610 ^b^ (515) ^c^					
Expt (MB^+^) ^e^		664 ^b^ (655) ^f^					

^a^ DFT/def2-TZVP. ^b^ In water solvent. ^c^ In benzene solvent. ^d^ Ref. [12]. ^e^ Ref. [20]. ^f^ In EtOH solvent.

**Table 3:** Emission λ (nm) and f-values of the S_1_→S_0_ and S_2_→S_0_ de-excitation of calculated structures of TH_s_-Cl and MB_S,H_-Cl at the B3LYP/def2-TZVP level of theory in water solvent.

λ_1_	f	MO	λ_2_	f	MO
S_1_→S_0_			S_2_→S_0_		
TH-Cl_S_					
642	0.004	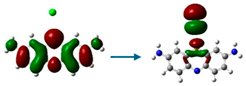	542	1.058	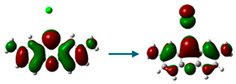
MB-Cl_S,H_					
666(657 ^a^)	0.843	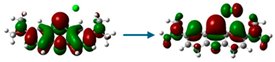	547(560 ^a^)	0.317	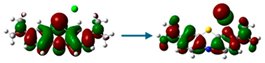

^a^ cLR value.

**Table 4:** Selected λ (nm) and dipole strength values (a.u.) of vibronic transitions of calculated minimum structures of TH and MB in solvents and gas phase at B3LYP/6-31G(d,p) method.

Molecule	Solvent	Excitation	λ	Dipole Strength	Expt
TH-Cl-W	water	|0> → |0>	582.0	6.177	599–560 ^a^
		|0> → |7^1^2^2^>	575.8	0.005456	
TH^+^	water	|0> → |0>	558.8	9.882	
		|0> → |6^1^>	553.5	1.034	
		|0> → |14^1^2^1^>	544.8	0.0216	
MB^+^	water	|0> → |0>	622.8	13.25	610–644 ^b^
		|0> → |5^1^>	618.7	1.699	
	chloroform	|0> → |0>	595.3	10.02	
		|0> → |5^1^>	591.5	1.56	
	gas phase	|0> → |0>	534.4	0.9489	
		|0> → |5^1^>	531.5	0.7328	
		|0> → |27^2^5^2^>	502.0	0.001371	
		|0> → |43^1^14^2^>	499.1	0.001759	

^a^ References [16,20], depending on pH values. ^b^ References [12,20], depending on pH values.

**Table 5:** Vis absorption main peaks, λ (nm), f-values and vibronic transitions of calculated minimum structures of methylene blue and thionine in water solvent.

Molecule	λ ^a^	f ^a^	Vibronic ^b^	Expt ^c^
**TH-Cl_H_/TH-Cl_S_**	495/489	0.651/0.673	576	560–599
**TH-Cl-W/(TH-Cl-W)_2_**	503/481	0.598/1.067	582	
**MB-Cl_S,H_/MB-Cl_N_**	530/520	0.839/0.805	622	610–644
**MB-Cl-W/(MB-Cl-W)_2_**	539(565)/502	0.881(0.027)/0.538		

^a^ B3LYP/def2-TZVP. ^b^ B3LYP/6-31G(d,p). ^c^ References [12,16,20], depending on pH values.

**Table 6:** Vertical triplet excitation energies ΔE (eV) of S_0_ → T_1_ excitations of calculated minimum structures of methylene blue and thionine in water solvent at B3LYP/def2-TZVP method.

Molecule	ΔE	λ	^3^O_2_(^3^Σ^−^_g_) → ^1^O_2_(^1^Δ_g_)
**TH^+^-W/TH-Cl_H_**	1.44/0.86	861/1440	0.98
**TH-Cl-W/(TH-Cl-W)_2_**	1.12/1.11	1110/1114	
**MB-Cl_S,H_/MB-Cl_N_**	1.30/1.32	955/939	
**MB-Cl-W/(MB-Cl-W)_2_**	1.20/1.19	1035/1046	(1.1 ^a^)

^a^ A good type II photosensitizer should have a T_1_ state lying >0.98 [59] >1.13 eV [60].

## Data Availability

The original contributions presented in this study are included in the article/Supplementary Materials. Further inquiries can be directed to the corresponding author.

## Supplementary Materials

The following supporting information can be downloaded at: https://www.mdpi.com/article/10.3390/molecules31213842/s1. Geometries of the calculated structures. Absolute Gibbs energies of the formation of the dimers (TH-Cl-W)_2_. ESPs. Natural Transition Orbitals. Absorption ΔE (eV), λ (nm) values and main excitation of S_0_ → T_1_, S_0_ → T_2_, and S_0_ → T_3_ excitations. Absolute energies of thionine structures. Figure S1. (a) Initial geometries of two TH-Cl-W molecules placed initially perpendicular and (b) the optimized system. Table S1. Absolute Gibbs energies in Hartree and ΔG in kcal/mol of the formation of the dimers (TH-Cl-W)_2_. Figure S2. ESP of TH-Cl-W, MB-Cl-W, (TH-Cl-W)_2_ and (MB-Cl-W)_2_ at B3LYP/def2-TZVP. Figure S3. Natural Transition Orbitals TH-Cl-W, MB-Cl-W, (TH-Cl-W)_2_ and (MB-Cl-W)_2_ at B3LYP/def2-TZVP. Table S2: Absorption ΔE (eV), λ (nm) values and main excitation of S_0_ → T_1_, S_0_ → T_2_, and S_0_ → T_3_ excitations at B3LYP/def2-TZVP method. Table S3. Absolute energies of thionine structures.

## References

[1] Chen J., Keltner L., Christophersen J., Zheng F., Krouse M., Singhal A., Wang S. (2002). New Technology for Deep Light Distribution in Tissue for Phototherapy. Cancer J..

[2] Dougherty T.J., Gomer C.J., Henderson B.W., Jori G., Kessel D., Korbelik M., Moan J., Peng Q. (1998). Photodynamic Therapy. J. Natl. Cancer Inst..

[3] Josefsen L.B., Boyle R.W. (2008). Photodynamic Therapy and the Development of Metal-Based Photosensitisers. Met. Based Drugs.

[4] Kwiatkowski S., Knap B., Przystupski D., Saczko J., Kędzierska E., Knap-Czop K., Kotlińska J., Michel O., Kotowski K., Kulbacka J. (2018). Photodynamic therapy–mechanisms, photosensitizers and combinations. Biomed. Pharmacother..

[5] Benov L. (2015). Photodynamic therapy: Current status and future directions. Med. Princ. Pract..

[6] Brown S.B., Brown E.A., Walker I. (2004). The present and future role of photodynamic therapy in cancer treatment. Lancet Oncol..

[7] Tzeli D., Petsalakis I.D. (2019). Physical insights into molecular sensors, molecular logic gates and on photosensitizers in photodynamic therapy. J. Chem..

[8] Zhou Z., Zhang L., Zhang Z., Liu Z. (2021). Advances in photosensitizer-related design for photodynamic therapy. Asian J. Pharm. Sci..

[9] Gualdesi M.S., Vara J., Aiassa V., Alvarez Igarzabal C.I., Ortiz C.S. (2020). Thionine in the design of new photosensitizers: Bromination and vehiculization in polymeric nanoparticles. J. Mol. Liq..

[10] Porcal G.V., Arbeloa E.M., Bertolotti S.G., Previtali C.M. (2017). Photophysics of thionine in AOT and BHDC reverse micelles. Quenching of the triplet state by aliphatic amines studied by transient absorption spectroscopy. J. Photochem. Photobiol. A Chem..

[11] Wisniak J. (2024). Charles Lauth: Dyes and other compounds. Educ. Quim..

[12] Ganeev R.A., Zvyagin A.I., Ovchinnikov O.V., Smirnov M.S. (2018). Peculiarities of the nonlinear optical absorption of Methylene blue and Thionine in different solvents. Dyes Pigments.

[13] Kamat P.V., Lichtin N.N. (1981). Electron-transfer in the quenching of the protonated triplet methylene-blue by ground-state molecules of the dye. J. Phys. Chem..

[14] Chen J., Cesario T.C., Rentzepis P.M. (2011). Effect of pH on methylene blue transient states and kinetics and bacteria photoinactivation. J. Phys. Chem. A.

[15] Lee S.-K., Mills A. (2003). Novel photochemistry of leuco-methylene blue. Chem. Commun..

[16] Nicotra V.E., Mora M., Iglesias R., Baruzzi A. (2008). Spectroscopic characterization of thionine species in different media. Dyes Pigments.

[17] Harris F., Chatfield L.K., Phoenix D.A. (2005). Phenothiazinium based photosensitisers-photodynamic agents with a multiplicity of cellular targets and clinical applications. Curr. Drug Targets.

[18] Cwalinski T., Polom W., Marano L., Roviello G., D’Angelo A., Cwalina N., Matuszewski M., Roviello F., Jaskiewicz J., Polom K. (2020). Methylene Blue-Current Knowledge, Fluorescent Properties, and Its Future Use. J. Clin. Med..

[19] Melgoza D., Hernandez-Ramirez A., Peralta-Hernandez J.M. (2009). Comparative efficiencies of the decolourisation of Methylene Blue using Fenton’s and photo-Fenton’s reactions. Photochem. Photobiol. Sci..

[20] Palit S.R., Saxena G.K. (1966). Absorption Spectra of Methylene Blue in Benzene. Nature.

[21] Ballard R.E., Park C.H. (1970). Optical absorption bandshapes of Acridine Orange, Thionine, and Methylene Blue in monomeric and dimeric states. J. Chem. Soc. A Inorg. Phys. Theor..

[22] Dean J.C., Oblinsky D.G., Rather S.R., Scholes G.D. (2016). Methylene Blue Exciton States Steer Nonradiative Relaxation: Ultrafast Spectroscopy of Methylene Blue Dimer. J. Phys. Chem. B.

[23] Kofler B., Romani A., Pritz C., Steinbichler T.B., Schartinger V.H., Riechelmann H., Dudas J. (2018). Photodynamic Effect of Methylene Blue and Low-Level Laser Radiation in Head and Neck Squamous Cell Carcinoma Cell Lines. Int. J. Mol. Sci..

[24] Thompson B.J., Kumar A., Huxter V.M. (2024). Concentration-dependent aggregation of methylene blue acting as a photoredox catalyst. Phys. Chem. Chem. Phys..

[25] Whang T.-J., Huang H.-Y., Hsieh M.-T., Chen J.-J. (2009). Laser-Induced Silver Nanoparticles on Titanium Oxide for Photocatalytic Degradation of Methylene Blue. Int. J. Mol. Sci..

[26] Olesund A., Gray V., Mårtensson J., Albinsson B. (2021). Diphenylanthracene Dimers for Triplet–Triplet Annihilation Photon Upconversion: Mechanistic Insights for Intramolecular Pathways and the Importance of Molecular Geometry. J. Am. Chem. Soc..

[27] Khan I., Saeed K., Zekker I., Zhang B., Hendi A.H., Ahmad A., Ahmad S., Zada N., Ahmad H., Ali Shah L. (2022). Review on Methylene Blue: Its Properties, Uses, Toxicity and Photodegradation. Water.

[28] Rodriguez-Serrano A., Daza M.C., Doerr M., Marian C.M. (2012). A quantum chemical investigation of the electronic structure of thionine. Photochem. Photobiol. Sci..

[29] Kostjukova L.O., Leontieva S.V., Kostjukov V.V. (2021). TD-DFT/DFT study of thionine in aqueous solution: Vibronic absorption spectrum and electronic properties. Optik.

[30] Kostjukova L.O., Leontieva S.V., Kostjukov V.V. (2021). Vibronic absorption spectrum and electronic properties of methylene blue in aqueous solution: TD-DFT study. J. Mol. Liq..

[31] Becke A.D. (1993). Density-functional thermochemistry. III. The role of exact exchange. J. Chem. Phys..

[32] Lee C., Yang W., Parr R.G. (1988). Development of the Colle-Salvetti correlation-energy formula into a functional of the electron density. Phys. Rev. B.

[33] Curtiss L.A., McGrath M.P., Blaudeau J.P., Davis N.E., Binning R.C., Radom L. (1995). Extension of Gaussian-2 theory to molecules containing third-row atoms Ga–Kr. J. Chem. Phys..

[34] Weigend F., Ahlrichs R. (2005). Balanced basis sets of split valence, triple zeta valence and quadruple zeta valence quality for H to Rn: Design and assessment of accuracy. Phys. Chem. Chem. Phys..

[35] Weigend F. (2006). Accurate Coulomb-fitting basis sets for H to Rn. Phys. Chem. Chem. Phys..

[36] Adamo C., Barone V. (1999). Toward reliable density functional methods without adjustable parameters: The PBE0 model. J. Chem. Phys..

[37] Perdew J.P., Burke K., Ernzerhof M. (1996). Generalized Gradient Approximation Made Simple. Phys. Rev. Lett..

[38] Chai J.D., Head-Gordon M. (2008). Long-range corrected hybrid density functionals with damped atom-atom dispersion corrections. Phys. Chem. Chem. Phys..

[39] Zhao Y., Truhlar D.G. (2008). The M06 suite of density functionals for main group thermochemistry, thermochemical kinetics, noncovalent interactions, excited states, and transition elements: Two new functionals and systematic testing of four M06-class functionals and 12 other functionals. Theor. Chem. Acc..

[40] Miertuš S., Scrocco E., Tomasi J. (1981). Electrostatic interaction of a solute with a continuum. A direct utilizaion of AB initio molecular potentials for the prevision of solvent effects. Chem. Phys..

[41] Tomasi J., Mennucci B., Cammi R. (2005). Quantum Mechanical Continuum Solvation Models. Chem. Rev..

[42] Chung W., Sameera W.M.C., Ramozzi R., Page A.J., Hatanaka M., Petrova G.P., Harris T.V., Li X., Ke Z., Liu F. (2015). The ONIOM Method and Its Applications. Chem. Rev..

[43] Stewart J.J.P. (2007). Optimization of parameters for semiempirical methods V: Modification of NDDO approximations and application to 70 elements. J. Mol. Model..

[44] Tzeliou C.E., Mermigki M.A., Tzeli D. (2022). Review on the QM/MM Methodologies and Their Application to Metalloproteins. Molecules.

[45] Tzeli D., Theodorakopoulos G., Petsalakis I.D., Ajami D., Rebek J. (2012). Conformations and Fluorescence of Encapsulated Stilbene. J. Am. Chem. Soc..

[46] Skarmoutsos I., Tzeli D., Petsalakis I.D. (2023). Hydration structure and dynamics, ultraviolet-visible and fluorescence spectra of caffeine in ambient liquid water. A combined classical molecular dynamics and quantum chemical study. J. Mol. Liq..

[47] Morgante P., Peverati R. (2023). Comparison of the Performance of Density Functional Methods for the Description of Spin States and Binding Energies of Porphyrins. Molecules.

[48] Jacquemin G.D., Planchat A., Adamo C., Mennucci B. (2012). TD-DFT Assessment of Functionals for Optical 0–0 Transitions in Solvated Dyes. J. Chem. Theory Comput..

[49] Kolokytha C., Sinani A., Manouras T., Angelakos E., Argitis P., Lathiotakis N.N., Riziotis C., Tzeli D. (2025). Photophysical Investigation of Dyes and Dye-PMMA Systems: Insights into Absorption, Emission, and Charge Transfer Mechanisms. J. Phys. Chem. A.

[50] Avramopoulos A., Reis H., Tzeli D., Zalesny R., Papadopoulos M.G. (2023). Photoswitchable Molecular Units with Tunable Non-Linear Optical Activity: A Theoretical Investigation. Molecules.

[51] Caricato M., Mennucci B., Tomasi J., Ingrosso F., Cammi R., Corni S., Scalmani G. (2006). Formation and relaxation of excited states in solution: A new time dependent polarizable continuum model based on time dependent density functional theory. J. Chem. Phys..

[52] Guido C.A., Caprasecca S. (2016). How to Perform Corrected Linear Response Calculations in G09.

[53] Santoro F., Lami A., Improta R., Bloino J., Barone V. (2008). Effective Method for the Computation of Optical Spectra of Large Molecules at Finite Temperature Including the Duschinsky and Herzberg–Teller Effect: The *Q_x_* Band of Porphyrin as a Case Study. J. Chem. Phys..

[54] Baiardi A., Bloino J., Barone V. (2013). General Time Dependent Approach to Vibronic Spectroscopy Including Franck-Condon, Herzberg-Teller, and Duschinsky Effects. J. Chem. Theory Comput..

[55] Frisch M.J., Trucks G.W., Schlegel H.B., Scuseria G.E., Robb M.A., Cheeseman J.R., Scalmani G., Barone V., Mennucci B., Petersson G.A. (2016). Gaussian 16, Revision C.01.

[56] Tzeli D., Petsalakis I.D., Theodorakopoulos G. (2011). Electronic structure and absorption spectra of supramolecular complexes of a fullerene crown ether with a π-extended TTF derivative. Phys. Chem. Chem. Phys..

[57] Selvam S., Sarkar I. (2017). Bile salt induced solubilization of methylene blue: Study on methylene blue fluorescence., properties and molecular mechanics calculation. J. Pharm. Anal..

[58] Arulraj A.D., Devasenathipathy R., Chen S.-M., Vasantha W.S., Wang S.-F. (2015). Highly selective and sensitive fluorescent chemosensor for femtomolar detection of silver ion in aqueous medium. Sens. Bio Sens. Res..

[59] Drzewiecka-Matuszek A., Rutkowska-Zbik D. (2021). Application of TD-DFT Theory to Studying Porphyrinoid-Based Photosensitizers for Photodynamic Therapy: A Review. Molecules.

[60] Rettig I.D., McCormick T.M. (2021). Enrolling reactive oxygen species in photon-to-chemical energy conversion: Fundamentals, technological advances, and applications. Adv. Phys. X.

