# Supplementary material for: Photophysical Properties of Thionine and Methylene Blue Photosensitizers: A TD-DFT Study of Monomers, Salts, Hydrates, and Dimers
Source: Molecules. 2026 Oct 24;31(21):3842. doi: 10.3390/molecules31213842 (PMC13765288; doi:10.3390/molecules31213842)
Supplement: Supplementary file 1 [file molecules-31-03842-s001.zip › molecules-4562537-supplementary.pdf]

Supporting Information

Photophysical Properties of Thionine and Methylene Blue Photosensitizers: A TD-DFT Study of Monomers, Salts, Hydrates, and Dimers

Christina Eleftheria Tzeliou [1], Eleni Dimou [1], Demeter Tzeli [1, 2*]

[1] *Laboratory of Physical Chemistry, Department of Chemistry, National and Kapodistrian University of Athens, Panepistimiopolis Zografou, Athens 157 84, Greece*
[2] *Theoretical and Physical Chemistry Institute, National Hellenic Research Foundation, 48 Vassileos Constantinou Ave., Athens 116 35, Greece*

Computational details:

Absorption Spectra: The standard UV-Vis absorption spectrum was simulated by convoluting the vertical electronic excitation energies and oscillator strengths using a Gaussian broadening function with a standard deviation (σ) of 0.4 eV (corresponding to a HWHM of ≈ 0.236 eV or 1,900 cm). The same bandwidth parameter was applied uniformly across all investigated molecular systems and solvents. The calculated vertical excitation energies and oscillator strengths (f) were convoluted using a standard Gaussian line-shape function with a standard deviation (σ) of 0.4 eV to simulate the continuous UV-Vis absorption curves in units of molar extinction coefficient (M^{-1} cm^{-1}).

(TH-Cl-W)$_2$: Different initial structures of (TH-Cl-W)$_2$ were investigated, including also structures where the TH-Cl-W molecules were placed perpendicular to each other, see Figure S1a (left structure). Geometry optimization of the system optimization led the two molecules to be placed in parallel, as expected, see Figure 1b.

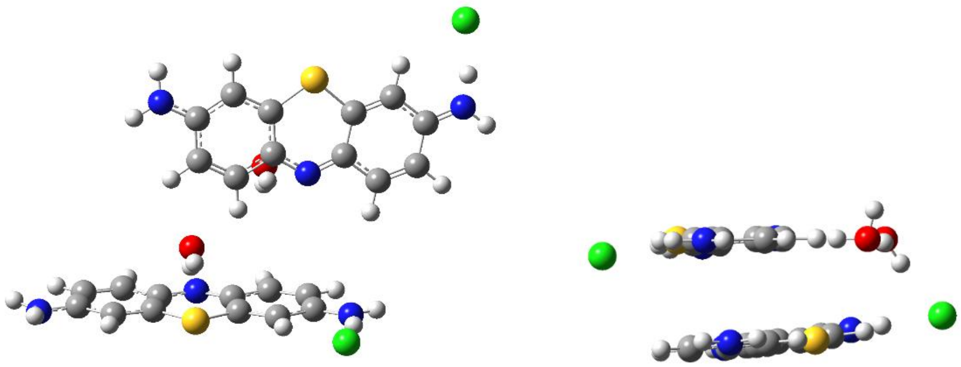

(a) (b)

Figure S1. (a) Initial geometries of two TH-Cl-W molecules placed initially perpendicular and (b) the optimized system

Table S1: Absolute Gibbs energies in Hartree and ΔG in kcal/mol of the formation of the dimers (TH-Cl-W)$_2$

	**(TH-Cl-W)$_2$**	**ΔG**
(TH-Cl-W)$_2$-1	-3123.998104	**-9.11**
(TH-Cl-W)$_2$-2	-3123.990110	**-4.11**
TH-Cl-W	-1561.991791	

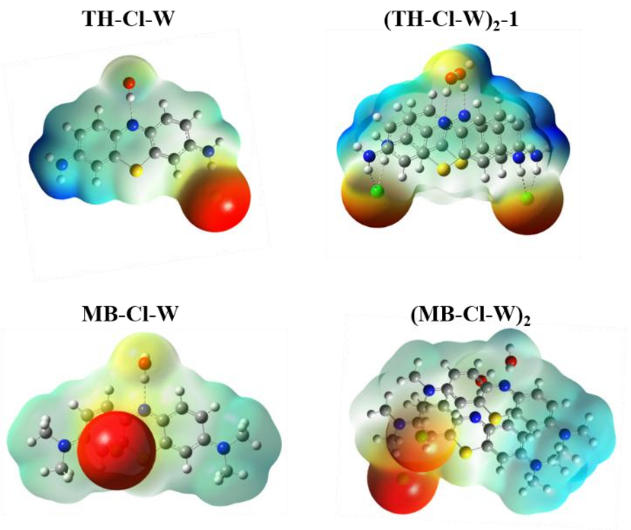

Figure S2. ESP of TH-Cl-W, MB-Cl-W, $(TH\text{-}Cl\text{-}W)_2$ and $(MB\text{-}Cl\text{-}W)_2$ at B3LYP/def2-TZVP.

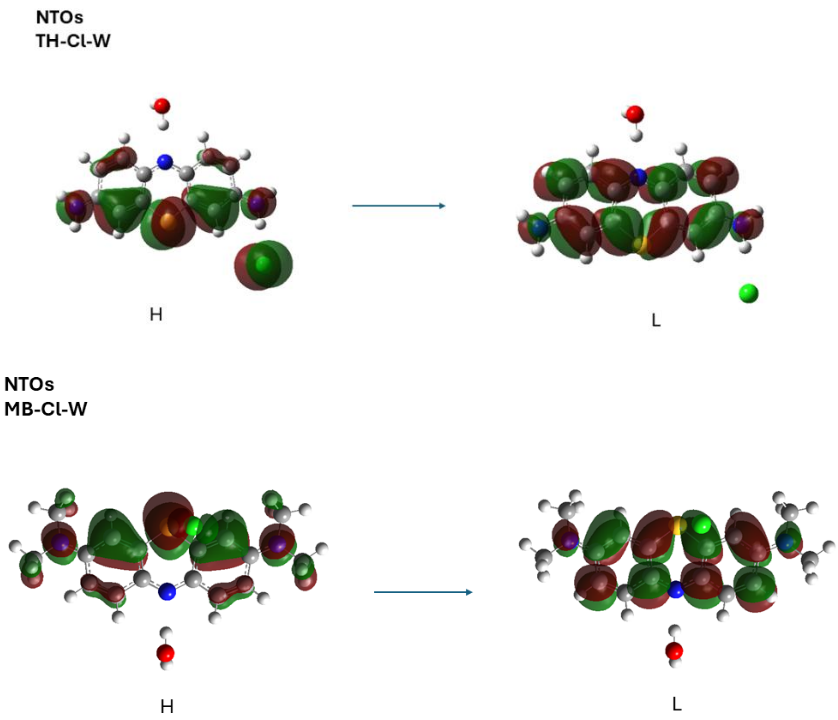

Figure S3. Natural Transition Orbitals TH-Cl-W, MB-Cl-W, $(TH\text{-}Cl\text{-}W)_2$ and $(MB\text{-}Cl\text{-}W)_2$ at B3LYP/def2-TZVP.

Table S2: Absorption ΔE (eV), λ (nm) values and main excitation of $S_0 \rightarrow T_1$, $S_0 \rightarrow T_2$, and $S_0 \rightarrow T_3$ excitations at B3LYP/def2-TZVP method.

**Molecule**	**State**	**ΔE (eV)**	**λ (nm)**	**Main Excitation**
**TH^+-W**	T_1	1.44	861.3	H → L
	T_2	1.94	640.1	H-1 → L
	T_3	2.78	447.4	H-2 → L
**TH-Cl_H**	T_1	0.86	1440.2	H → L
	T_2	0.95	1300.2	H-1 → L
	T_3	1.17	1061.7	H-2 → L
**$(TH\text{-}Cl\text{-}W)_2$-1**	T_1	1.11	1113.9	H → L
	T_2	1.17	1057.6	H → L+1
	T_3	2.04	608.7	H-2 → L
**MB-$Cl_{S,H}$**	T_1	1.30	955.4	H → L
	T_2	1.85	669.5	H-1 → L
	T_3	2.43	509.8	H-2 → L
**MB-Cl_N**	T_1	1.32	939.3	H → L
	T_2	1.89	657.6	H-1 → L
	T_3	2.22	556.3	H-2 → L
**MB-Cl-W**	T_1	1.20	1034.5	H → L
	T_2	1.78	695.9	H-1 → L
	T_3	2.19	567.4	H-2→ L
**$(MB\text{-}Cl\text{-}W)_2$**	T_1	1.19	1046.3	H → L
	T_2	1.21	1023.5	H-1 → L
	T_3	1.70	729.5	H → L+1

Geometries and absolute energies

Table S3: Absolute energies of thionine structures

**Charge**	**2S+1**		**Functional**	**Basis Set**	**-E** (hartree)
0	2	TH-W	B3LYP	6-31G(d,p)	1025.661089
1	1	TH^{+}-W	B3LYP	6-31G(d,p)	1025.461872
0	1	$TH-Cl_{H}$	B3LYP	6-31G(d,p)	1485.927145
0	1	TH-Cl-W	B3LYP	6-31G(d,p)	1562.346107
0	1	TH-Cl-W	B3LYP	Def2TZVP	1562.664550
0	1	TH-Cl-W	PBE1PBE	Def2TZVP	1561.522583
0	1	TH-Cl-W	wB97XD	Def2TZVP	1562.368905
0	1	TH-Cl-W	m062x	Def2TZVP	1562.296344
0	1	$(TH-Cl-W)_2$-1	wB97XD	6-31G(d,p)	3124.362134
0	1	$(TH-Cl-W)_2$-2	wB97XD	6-31G(d,p)	3124.293010
0	1	$TH-Cl_S$	B3LYP	Def2TZVP	1486.145828
0	1	$TH-Cl_S@W_{61}$	B3LYP	6-31G(d,p)	1491.902997
0	1	$TH-Cl_S@W_{61}$	B3LYP	Def2TZVP	1492.140089
0	1	$TH-Cl_S@W_{61}$	wB97XD	Def2TZVP	1491.845681
0	1	$TH-Cl_S@W_{61}$	PBE1PBE	Def2TZVP	1491.078205
0	1	$TH-Cl_S@W_{61}$	m062x	Def2TZVP	1491.789578

- **TH-W (B3LYP/6-31G(d, p))**

0 2

C	3.79490500	-0.57434300	0.00864000
C	2.65134700	-1.38748100	-0.01454200
C	1.37609300	-0.82718700	-0.01448500
C	1.19195900	0.58512900	0.01182000
C	2.36760100	1.38417900	0.03419700
C	3.62874800	0.82945100	0.03158700
C	-1.19335500	0.58741500	0.01174700
C	-1.38035800	-0.82452600	-0.01449400
C	-2.65675100	-1.38224000	-0.01451300
H	-2.77302400	-2.46317600	-0.03750700
C	-3.79870000	-0.56686200	0.00857500
C	-3.62975300	0.83660300	0.03136000
C	-2.36743200	1.38865800	0.03398100

H	2.76547100	-2.46864300	-0.03761400
H	2.22514500	2.46046700	0.05894600
H	4.50567900	1.47113300	0.04567300
H	-4.50537200	1.48006500	0.04527000
H	-2.22301500	2.46467000	0.05848600
N	-5.06977400	-1.12289400	-0.04616300
H	-5.15054500	-2.08402800	0.25375200
H	-5.82111500	-0.54192200	0.29761900
N	-0.00012300	1.23908600	0.02050600
S	-0.00322900	-1.93556900	-0.04780600
N	5.06485400	-1.13288500	-0.04609600
H	5.81737200	-0.55337600	0.29757600
H	5.14376800	-2.09419700	0.25373400
O	0.02073700	4.09510300	0.02342700
H	0.02243100	4.24688400	-0.92999100
H	0.00845100	3.11448100	0.09057300

- **TH^{+}-W (B3LYP/6-31G(d, p))**

1 1

C	-3.70254700	-0.71958800	0.01184700
C	-2.53140300	-1.51240900	-0.00176400
C	-1.28576900	-0.91230900	-0.01099100
C	-1.15496100	0.52576800	-0.01162600
C	-2.36182500	1.30404000	0.00195000
C	-3.58739800	0.70840900	0.01451600
C	1.20853900	0.61498300	-0.01012400
C	1.45504200	-0.80422500	-0.01067300
C	2.74617200	-1.30370700	-0.00223800
H	2.91972400	-2.37578400	-0.00342600
C	3.85075400	-0.42301100	0.01176300
C	3.62163800	0.99102400	0.01695100

C	2.34939400	1.48067500	0.00565400
H	-2.62064200	-2.59478200	-0.00162400
H	-2.24807500	2.38616800	0.01387000
H	-4.49037200	1.31077300	0.03034000
H	4.47190600	1.66537900	0.03268900
H	2.16036300	2.54876000	0.01690100
N	5.11152100	-0.89671800	0.02171600
H	5.30417800	-1.88656000	0.02347700
H	5.90339200	-0.27262100	0.03807800
N	0.00218800	1.19232100	-0.01846600
S	0.12753100	-1.94077600	-0.02719800
N	-4.92003500	-1.29245400	0.02367800
H	-5.75882600	-0.73259500	0.04082900
H	-5.03376500	-2.29457800	0.02817800
O	-0.82202800	4.09549000	0.08951200
H	-0.30371500	3.28062500	-0.01082600
H	-0.76996300	4.53377800	-0.76950200

- **TH-Cl-W (B3LYP/6-31G(d, p) in water solvent)**

0 1

C	3.02071600	0.99525700	0.00933600
C	2.22087300	-0.18082000	0.00193700
C	0.84689500	-0.08726500	0.00583800
C	0.18757100	1.19973300	0.01768800
C	1.01982300	2.37033100	0.02432300
C	2.37717600	2.28239100	0.02042500
C	-2.04297100	0.40893600	0.01418900
C	-1.74804900	-0.99819100	0.00216600
C	-2.76374900	-1.93702700	-0.00525900
H	-2.53338200	-2.99773200	-0.01416200
C	-4.11711000	-1.52440000	-0.00180200

C	-4.42380100	-0.12510600	0.00940800
C	-3.41929500	0.79781200	0.01694700
H	2.72325700	-1.14328000	-0.00685400
H	0.51373000	3.33018100	0.03271000
H	2.99072800	3.17733200	0.02549200
H	-5.46294400	0.18636000	0.01166500
H	-3.63051000	1.86212800	0.02536200
N	-5.11268000	-2.42758400	-0.00932400
H	-4.92582200	-3.41879300	-0.01662200
H	-6.07864800	-2.13735200	-0.00621000
N	-1.12939700	1.39088400	0.02221200
S	-0.09120900	-1.56780300	-0.00281500
N	4.35051700	0.89870700	0.00575000
H	4.81970300	-0.02325800	-0.00159100
H	4.92060700	1.73229300	0.01115200
Cl	5.60010600	-2.02351100	-0.01772700
O	-2.10478100	4.14018000	0.03903300
H	-1.77820200	3.21824400	0.05728700
H	-2.17026700	4.33300900	-0.90574100

- **TH-Cl$_H$ (B3LYP/6-31G(d, p))**

0 1

C	-3.03544800	0.99082900	0.00079400
C	-2.11442600	-0.10883200	0.00438900
C	-0.76781700	0.12497400	0.00493700
C	-0.22406900	1.47996600	0.00113100
C	-1.18007600	2.56414300	-0.00167400
C	-2.51707400	2.34245300	-0.00176400
C	2.06054700	0.90491400	0.00051600
C	1.90487000	-0.51359700	0.00571600
C	3.01502100	-1.35217700	0.00410600

H	2.88001800	-2.43035900	0.00959300
C	4.31514700	-0.81733500	-0.00086400
C	4.48305400	0.59461500	-0.00515800
C	3.38525300	1.41811000	-0.00463700
H	-2.54806800	-1.10981900	0.00595400
H	-0.76558000	3.56649200	-0.00409800
H	-3.21994200	3.17058900	-0.00415600
H	5.48544000	1.01217500	-0.00584800
H	3.49221500	2.49750200	-0.00919400
N	5.40679800	-1.63745200	0.02958400
H	5.29637900	-2.62604200	-0.13004400
H	6.32125700	-1.25571400	-0.15077500
N	1.04945300	1.81252000	-0.00101400
S	0.31796700	-1.25825100	0.01132300
N	-4.32961800	0.74022200	-0.00015800
H	-4.67845300	-0.30066900	-0.00185600
H	-4.98636100	1.50995000	-0.00357200
Cl	-4.89415900	-2.12220600	-0.00770600

- **TH-Cl$_S$ (B3LYP/def2-TZVP)**

0 1

C	3.729713	-0.197408	-0.287585
C	2.599693	0.537714	-0.595778
C	1.321581	0.008340	-0.370975
C	1.149454	-1.293954	0.195459
C	2.339447	-2.024959	0.488117
C	3.574793	-1.506751	0.255837
C	-1.179891	-1.278402	0.206911
C	-1.340116	0.027045	-0.358437
C	-2.614206	0.568418	-0.582033
H	-2.700894	1.574690	-0.973784
C	-3.751324	-0.155052	-0.270081

C	-3.607450	-1.463672	0.281059
C	-2.377381	-1.995444	0.506587
H	2.695063	1.545296	-0.981742
H	2.213761	-3.011407	0.913865
H	4.460569	-2.085347	0.490504
H	-4.498371	-2.027285	0.532143
H	-2.260653	-2.982725	0.932962
N	-5.008871	0.368413	-0.430772
H	-5.119775	1.212358	-0.965397
H	-5.796872	-0.254940	-0.432991
N	-0.018618	-1.883848	0.458391
S	-0.004120	1.033501	-0.699023
N	4.994992	0.286025	-0.529903
H	5.094869	1.277967	-0.669996
H	5.758068	-0.139235	-0.030611
Cl	0.081279	2.917954	1.121819

- **$(TH\text{-}Cl\text{-}W)_2$-1 (ωB97XD/6-31G(d, p) in water solvent)**

0 1

C	-3.07281600	0.26747900	-1.82851900
C	-1.96151700	-0.60834900	-1.81441000
C	-0.68149400	-0.10436100	-1.76642500
C	-0.44310700	1.31335000	-1.70233100
C	-1.58685600	2.17683300	-1.73065900
C	-2.84779600	1.68425200	-1.79856300
C	1.90557000	1.21787900	-1.55353300
C	2.03292000	-0.21182000	-1.58183600
C	3.25885700	-0.82080800	-1.43913000
H	3.34751000	-1.90175600	-1.40334800
C	4.42500800	-0.04170600	-1.27737800
C	4.32315300	1.38757800	-1.30942400
C	3.10887500	1.98196600	-1.42906000

H	-2.14096800	-1.67861900	-1.83259200
H	-1.40637600	3.24523100	-1.68282900
H	-3.70173600	2.35228500	-1.80856200
H	5.22278100	1.98315600	-1.20216500
H	3.01739200	3.06193700	-1.41463300
N	5.60496900	-0.63849500	-1.08744700
H	5.62401700	-1.62755800	-0.82963700
H	6.42407200	-0.08436100	-0.89239000
N	0.75448100	1.88578200	-1.61190800
S	0.64244000	-1.22905400	-1.79590800
N	-4.31153800	-0.21726400	-1.86131600
H	-4.49810900	-1.22046100	-1.74395600
H	-5.10006100	0.41007700	-1.82855600
Cl	-4.94225200	-3.29714300	-1.11590100
O	0.33334900	4.65358200	-0.87982900
H	0.65450200	3.78275300	-1.17684100
H	1.00810300	5.28778000	-1.13713000
C	3.05705300	-0.28866400	1.88635300
C	1.93651000	-1.14526900	1.77197400
C	0.66285400	-0.62203000	1.75645800
C	0.44661600	0.79758600	1.82990700
C	1.59812400	1.64138300	1.95176600
C	2.85159200	1.12648300	1.99440800
C	-1.90203900	0.75931600	1.67721800
C	-2.05710300	-0.66249000	1.55724800
C	-3.29909100	-1.22564300	1.36514500
H	-3.41235100	-2.29444000	1.22045200
C	-4.44722700	-0.40740800	1.29849300
C	-4.31555100	1.00703200	1.48210700
C	-3.08668200	1.56030800	1.64509700
H	2.10199500	-2.21424100	1.68610800
H	1.41727600	2.71096900	2.00111800

H	3.71439100	1.77766900	2.08060100
H	-5.20257800	1.62946200	1.44448600
H	-2.95997900	2.63345500	1.73756800
N	-5.64522300	-0.95714000	1.06060000
H	-5.66953300	-1.87494900	0.61484500
H	-6.44133000	-0.35730200	0.90839200
N	-0.74020400	1.39648900	1.79589500
S	-0.68106800	-1.72017600	1.64421000
N	4.29308800	-0.78878500	1.89275400
H	4.47606400	-1.74959700	1.58428000
H	5.08147000	-0.16027400	1.90037700
Cl	4.98558500	-3.52329100	0.32964900
O	-0.34347400	4.35086700	1.82956400
H	-0.61967100	3.42259000	1.84166200
H	-0.09860300	4.50417600	0.89884800

- **$(TH\text{-}Cl\text{-}W)_2$-2 (ωB97XD/6-31G(d, p) in water solvent)**

0 1

C	1.89790600	1.76585000	-1.59402800
C	1.11764400	0.61922300	-1.89301200
C	-0.23885800	0.63386500	-1.68102300
C	-0.89344100	1.79856100	-1.13573500
C	-0.07861400	2.94439600	-0.84495800
C	1.25572800	2.94003300	-1.06757600
C	-3.06259000	0.89140000	-1.01336900
C	-2.74877400	-0.40849100	-1.52126400
C	-3.70580800	-1.40151200	-1.56831300
H	-3.45362900	-2.39235100	-1.93119700
C	-5.01382500	-1.14639900	-1.10960500
C	-5.35052800	0.16121400	-0.63703700
C	-4.40515300	1.13572000	-0.59487300
H	1.62512400	-0.25840200	-2.28350900

H	-0.56885400	3.80367900	-0.40301400
H	1.86096000	3.80721100	-0.82875500
H	-6.36133600	0.35671500	-0.29723600
H	-4.63784300	2.12314400	-0.21113200
N	-5.93464500	-2.11888900	-1.09331900
H	-5.72319600	-3.04546000	-1.42676500
H	-6.87663400	-1.93990800	-0.78537500
N	-2.18463400	1.88887100	-0.86367000
S	-1.15375800	-0.78258200	-2.10073200
N	3.21038100	1.73871500	-1.77609100
H	3.66921800	0.88868300	-2.13844900
H	3.77379600	2.54630000	-1.55904000
Cl	4.27046600	-1.01588100	-2.91265700
O	-2.98323900	4.10259000	0.73333700
H	-2.77457400	3.41996800	0.06674900
H	-2.96838900	4.93753000	0.25633200
C	3.99431800	0.54711600	1.21645900
C	3.23523600	-0.63435900	1.00848200
C	1.87846700	-0.62594800	1.20645100
C	1.19319700	0.57748900	1.62360000
C	1.98791100	1.76208100	1.81499700
C	3.32707300	1.75475300	1.62618300
C	-0.97644000	-0.33296900	1.72815100
C	-0.64425000	-1.66270200	1.33069300
C	-1.61743800	-2.63854000	1.22853500
H	-1.35811500	-3.64295000	0.90998700
C	-2.95960000	-2.33698100	1.53504800
C	-3.29963900	-1.01737900	1.96038000
C	-2.33959600	-0.05583200	2.03833800
H	3.75917700	-1.53136700	0.69315100
H	1.45871800	2.66292100	2.10836300
H	3.91378800	2.65478200	1.77262700

H	-4.33123300	-0.78683800	2.20062100
H	-2.58489000	0.95631900	2.34255700
N	-3.91466100	-3.27451800	1.43209900
H	-3.70378000	-4.20001600	1.09678500
H	-4.88263800	-3.05014500	1.59439800
N	-0.10287300	0.68091400	1.84365800
S	0.99381100	-2.09661000	0.92961100
N	5.30626700	0.53140500	1.03321300
H	5.79438200	-0.34680700	0.79997500
H	5.85290100	1.36375300	1.19214600
Cl	6.57773700	-2.33087000	0.52121100
O	-0.88881300	3.47010300	2.52121100
H	-0.75003800	2.53777400	2.29764200
H	-1.59319500	3.76674900	1.91891600

- **TH-Cl@W61 ONIOM(B3LYP/6-31G(d, p):PM6)**

0 1

O	3.847406	-3.283609	-4.351828
H	4.273654	-4.021705	-3.927581
H	4.158124	-2.478452	-3.910810
O	-2.067183	-5.278552	-1.802133
H	-1.541214	-5.292236	-2.607065
H	-1.438812	-5.426601	-1.067986
O	1.361451	-4.660184	-1.512030
H	1.091819	-5.550968	-1.232395
H	1.052828	-4.576641	-2.476200
O	-3.748250	-4.253509	3.192071
H	-3.914455	-3.260141	3.311852
H	-4.579606	-4.680504	3.134519
O	7.045676	1.979170	0.521358
H	7.083496	1.231277	1.151643

H	7.387718	1.674349	-0.316735
O	6.241812	-0.012895	2.138407
H	6.200980	0.049251	3.115191
H	5.359032	0.301336	1.871372
O	-2.388939	-2.226108	-4.581123
H	-2.616001	-2.719226	-5.357625
H	-2.508960	-1.277095	-4.762742
O	0.436186	-4.528988	4.556263
H	0.100022	-4.485874	3.651103
H	1.387525	-4.759053	4.491623
O	4.189587	-3.420219	-1.979734
H	4.598369	-3.342994	-1.103585
H	3.245180	-3.585961	-1.828839
O	-6.824691	-2.322485	-2.880514
H	-5.944143	-2.587685	-2.506327
H	-6.652580	-1.646155	-3.524515
O	-1.321662	4.473881	-3.788318
H	-1.366190	4.494577	-2.808724
H	-0.380864	4.482254	-4.024866
O	-2.404667	6.689112	2.471479
H	-1.833761	7.404439	2.717953
H	-2.799551	6.920642	1.574163
O	-5.598338	-1.101370	1.417658
H	-6.300401	-0.567006	1.799121
H	-4.965654	-1.320756	2.193178
O	4.592530	-2.477091	4.026844
H	4.276335	-1.628062	4.367650
H	5.043985	-2.299515	3.199134
O	-0.255357	-5.872475	0.277719
H	-0.440349	-6.775335	0.532424
H	-0.721463	-5.288069	1.008252
O	-3.208232	6.438071	0.034249

H	-3.844811	5.719092	0.274522
H	-2.425882	5.981891	-0.347292
O	1.452600	-3.520259	-5.729265
H	2.341191	-3.330627	-5.336647
H	1.065596	-2.634308	-5.896833
O	-0.192446	2.550833	3.295337
H	-1.071645	2.933053	3.139017
H	-0.409736	1.706908	3.901019
O	4.807168	3.516301	0.538136
H	5.641688	3.033229	0.325781
H	4.879800	3.785994	1.454301
O	-4.170003	0.409484	5.308579
H	-4.834972	1.062812	5.119370
H	-4.173921	0.255074	6.251153
O	6.371862	-2.906591	1.987957
H	6.870847	-2.082286	1.942394
H	6.874615	-3.531539	2.505309
O	-0.635898	-3.446733	-0.084079
H	-1.337004	-3.306898	-0.783388
H	0.054704	-3.982978	-0.567061
O	2.889074	4.325933	2.901792
H	2.396699	4.766960	3.617020
H	3.166234	5.004079	2.250517
O	-6.855548	-1.244598	-0.545993
H	-7.380316	-1.631420	-1.279972
H	-7.093662	-1.734413	0.277485
O	3.871487	-5.342714	-0.347134
H	4.180524	-5.606350	-1.210123
H	2.960903	-5.038046	-0.450697
O	4.770676	2.815369	-2.797704
H	3.794931	2.847799	-2.866305
H	5.016147	3.667711	-2.310825

O	3.125954	-4.875478	3.951653
H	3.775624	-4.154601	4.029544
H	2.717137	-4.749991	3.085512
O	1.423139	3.882576	4.911881
H	0.790388	3.327078	4.347176
H	0.939199	4.099991	5.702673
O	-6.802567	2.811586	0.958274
H	-6.957608	3.044431	0.039973
H	-6.228968	3.496969	1.333476
O	-2.805085	0.382061	-4.000287
H	-3.767807	0.560687	-3.902184
H	-2.446188	0.372134	-3.105459
O	3.431883	5.610007	0.598458
H	2.731217	5.029767	0.143181
H	3.133019	6.507269	0.511516
O	-4.426005	4.240538	0.983972
H	-4.339466	3.554590	0.307712
H	-3.662835	4.079418	1.639512
O	-1.527757	2.479366	-5.446151
H	-1.933569	1.680490	-5.062556
H	-1.947408	3.248939	-5.002706
O	-2.209219	-2.282296	-1.988903
H	-2.298914	-2.699078	-2.870103
H	-3.124609	-1.990248	-1.753546
O	-0.831166	-2.760619	5.771686
H	-1.202069	-2.958960	4.893017
H	-0.145873	-3.463236	5.891984
O	4.338531	0.141929	3.704545
H	4.365028	0.721526	4.515730
H	3.473664	0.347548	3.321157
O	-5.800646	4.338487	-1.041004
H	-5.632569	5.088860	-0.443085

H	-6.050267	4.717738	-1.883583
O	5.075795	5.075270	-1.432662
H	4.890708	4.908278	-0.444845
H	4.481506	5.764849	-1.689454
O	6.422859	-1.860783	-1.250021
H	6.150504	-2.633244	-1.762755
H	6.354689	-1.093651	-1.846394
O	-5.605426	0.547015	-3.888577
H	-6.088501	1.368346	-3.904830
H	-6.134232	-0.109856	-4.336580
O	1.321050	4.324793	-0.301793
H	1.280839	3.616696	0.493218
H	1.531237	3.741802	-1.079409
O	1.497626	-4.292620	1.646214
H	1.201982	-5.085520	1.163505
H	0.828788	-3.614588	1.465827
O	1.575682	-1.133519	-4.948168
H	1.671172	-1.352235	-4.012927
H	0.995395	-0.349135	-4.959818
O	0.377689	-0.524157	5.888090
H	-0.093406	-1.344868	5.471848
H	0.273074	-0.668321	6.827544
O	0.031890	-4.332693	-3.773115
H	-0.314602	-3.437190	-3.771359
H	0.607502	-4.365961	-4.626882
O	-4.741900	-2.482665	-1.211374
H	-5.329794	-1.884868	-0.596329
H	-4.475091	-3.234150	-0.678460
O	-0.092774	-0.869429	-3.239166
H	-0.706800	-1.580504	-2.975247
H	-0.519476	-0.054841	-2.983635
O	0.838920	3.353483	-5.258862

H	0.059032	2.694383	-5.157931
H	0.716716	3.731801	-6.132878
O	1.569691	2.460922	1.446660
H	0.717647	2.229872	1.940871
H	2.154133	2.896068	2.112775
O	-1.278989	-4.330921	2.152151
H	-1.388655	-3.467646	1.701230
H	-2.189711	-4.541485	2.507059
O	3.723685	2.359458	4.598048
H	3.737605	3.027100	3.892097
H	2.977636	2.607386	5.172268
O	-3.982967	-1.661506	3.397719
H	-2.874128	-1.297593	3.291484
H	-4.225378	-1.271317	4.236212
O	-2.460891	4.208474	2.736931
H	-1.971852	5.043742	2.474893
H	-2.909685	4.453808	3.558128
O	2.069593	3.378851	-2.856773
H	1.605569	2.538142	-3.129468
H	1.955068	3.987601	-3.595691
O	-1.279684	1.352889	5.140344
H	-0.658290	0.571527	5.436714
H	-2.079575	0.903293	4.807660
O	-0.895356	5.312814	-1.119225
H	-0.395857	6.068804	-1.428403
H	-0.241282	4.793144	-0.534527
C	-3.690187	1.128961	-0.622625
C	-2.834896	0.081804	-0.236815
C	-1.461139	0.208044	-0.362136
C	-0.862362	1.383302	-0.912358
C	-1.742122	2.427908	-1.293947
C	-3.103973	2.316811	-1.160534

C	1.386555	0.729802	-0.650445
C	1.132599	-0.569888	-0.040756
C	2.156146	-1.388716	0.378338
H	1.943449	-2.359548	0.828461
C	3.506776	-0.993841	0.214746
C	3.786547	0.284202	-0.384922
C	2.778548	1.098960	-0.794057
H	-3.265054	-0.804164	0.224603
H	-1.298815	3.344670	-1.684736
H	-3.755282	3.139627	-1.452149
H	4.829338	0.586443	-0.505530
H	2.984139	2.078116	-1.219813
N	4.519519	-1.758433	0.640629
H	4.386241	-2.632970	1.135512
H	5.492563	-1.520529	0.419648
N	0.476834	1.593903	-1.057667
S	-0.501247	-1.157658	0.146434
N	-5.031066	0.997816	-0.508935
H	-5.380253	0.132287	-0.076686
H	-5.612275	1.829030	-0.426278
Cl	-1.335738	-0.931847	3.295372
O	1.992867	1.372713	-4.445858
H	2.848341	0.945850	-4.365437
H	2.026254	1.972861	-5.223363
O	4.722805	-0.536127	-3.635618
H	5.577239	-0.141308	-3.366692
H	4.286877	0.105149	-4.183992
O	6.328748	0.975677	-2.156045
H	7.009136	1.553867	-2.494454
H	5.493738	1.555609	-2.120689
O	-8.207322	-1.198731	1.823817
H	-9.034160	-0.890390	1.465112

H	-8.373483	-1.533289	2.699695
O	-5.702756	-5.221268	-1.589672
H	-5.492510	-4.362546	-1.959524
H	-5.112153	-5.373758	-0.860751

- **MB-Cl$_{S,H}$ (ωB97XD/def2-TZVP in water solvent)**

0 1

C	-3.47206400	-0.77262700	-0.01932000
C	-2.40522900	0.15272600	-0.04490700
C	-1.10272500	-0.28651900	-0.02226600
C	-0.78202000	-1.68263000	0.02532600
C	-1.87833500	-2.59461800	0.04864400
C	-3.16111400	-2.17369700	0.02769400
C	1.54493600	-1.46277300	0.03064200
C	1.59397800	-0.03733800	-0.01682400
C	2.79537200	0.63649400	-0.03458800
H	2.78746400	1.71550600	-0.07101600
C	4.01794700	-0.06488700	-0.00596900
C	3.97336700	-1.49705100	0.04371000
C	2.79010400	-2.15157900	0.06035300
H	-2.59205800	1.22086200	-0.07162400
H	-1.64081500	-3.64961400	0.08356700
H	-3.95536800	-2.90363500	0.04555400
H	4.88995800	-2.06545000	0.06803300
H	2.75617800	-3.23245400	0.09722500
N	5.19150500	0.58138700	-0.02458200
N	0.43667800	-2.19374800	0.04913300
S	0.14839300	0.89940400	-0.05127700
N	-4.74353300	-0.36013200	-0.03880700
Cl	-2.42779200	3.78199100	0.12244300
C	6.44867500	-0.15097500	0.00516900
H	6.54446500	-0.80892100	-0.86053700

H	7.26855600	0.55926200	-0.01741900
H	6.53884300	-0.74615300	0.91571200
C	5.22844800	2.03316500	-0.07648300
H	4.74226500	2.40693500	-0.98030700
H	4.73664800	2.47084500	0.79501800
H	6.26234900	2.36180900	-0.08482400
C	-5.84314500	-1.31371100	-0.02170800
H	-5.81986500	-1.92702400	0.88066800
H	-6.78019900	-0.76707400	-0.03540200
H	-5.81549100	-1.96625300	-0.89632200
C	-5.05063900	1.05986200	-0.10764700
H	-6.12748400	1.19055800	-0.08611900
H	-4.66161300	1.50052800	-1.02778800
H	-4.61354800	1.60034900	0.73331100

- **MB-Cl$_N$ (ωB97XD/def2-TZVP in water solvent)**

0 1

C	3.68299600	0.19157900	-0.39249500
C	2.52919900	0.96488200	-0.14763300
C	1.27373400	0.42713400	-0.31098300
C	1.09281600	-0.92532400	-0.73941000
C	2.27269600	-1.68569500	-0.98695400
C	3.50880300	-1.16637500	-0.82303700
C	-1.24253200	-0.94652300	-0.69265900
C	-1.43123200	0.40031500	-0.25673700
C	-2.69042900	0.91621000	-0.04612700
H	-2.78640700	1.93910700	0.28520900
C	-3.83865900	0.12503200	-0.25464800
C	-3.65560600	-1.22686200	-0.69654000
C	-2.41584700	-1.72537500	-0.90252300
H	2.61977900	1.98947200	0.17963300
H	2.14043100	-2.70970300	-1.31019300

H	4.37084600	-1.78442600	-1.01941000
H	-4.51264900	-1.85901800	-0.86860400
H	-2.27792200	-2.74590000	-1.23446400
N	-5.06788100	0.61493900	-0.04741100
N	-0.07105400	-1.52735200	-0.91485600
S	-0.08191400	1.43447200	0.01117300
N	4.90898700	0.70200000	-0.22933300
Cl	0.68948100	-1.35831100	2.97109600
C	-5.24553700	1.98769200	0.39543500
H	-6.30586900	2.19244200	0.49882600
H	-4.76761100	2.15376400	1.36338000
H	-4.82940800	2.69135700	-0.32876400
C	-6.24761900	-0.21117500	-0.25716800
H	-6.32422400	-0.53644500	-1.29633100
H	-6.23525200	-1.08957100	0.39041500
H	-7.13052200	0.37257200	-0.01870700
C	5.07937200	2.07903400	0.20441500
H	6.13908900	2.30108400	0.27114400
H	4.62706300	2.77321200	-0.50696400
H	4.63179900	2.23909400	1.18759100
C	6.09520600	-0.10432500	-0.47898500
H	6.12351600	-0.97776100	0.17457700
H	6.13667900	-0.43529200	-1.51818500
H	6.97558400	0.49730400	-0.27906100

- **MB-Cl-W (B3LYP/def2-TZVP in water solvent)**

0 1

C	3.93220000	-0.24603500	-0.24213400
C	2.76908200	-1.02992400	-0.43691000
C	1.51508900	-0.45962600	-0.41079200
C	1.33910600	0.94682000	-0.18593800

C	2.52279600	1.71824800	0.00771400
C	3.76081500	1.16121600	-0.01699200
C	-1.01395900	0.98249200	-0.30338500
C	-1.20872900	-0.41901300	-0.54640900
C	-2.46978000	-0.95271800	-0.69274600
H	-2.56890500	-2.01200600	-0.87050400
C	-3.62510400	-0.13499900	-0.60519300
C	-3.43316400	1.26936400	-0.36799300
C	-2.18760400	1.78947600	-0.22563100
H	2.85482800	-2.09192000	-0.60745100
H	2.40373600	2.77981100	0.17848500
H	4.62263500	1.79110700	0.13530500
H	-4.28596000	1.92506900	-0.29777200
H	-2.05383500	2.84757200	-0.04450200
N	-4.86670800	-0.65223800	-0.73794700
N	0.16624700	1.57909800	-0.14454000
S	0.14634900	-1.49533000	-0.66149900
N	5.15975800	-0.79601200	-0.26643400
Cl	-1.47067600	-0.98041000	3.65320100
O	0.18852900	4.50623100	0.29424700
H	0.22972500	4.90372600	-0.58279500
H	0.18141100	3.54192600	0.13497800
C	-6.05383900	0.19784700	-0.64435600
H	-6.04681800	0.97115300	-1.41404200
H	-6.93507300	-0.41795300	-0.78763200
H	-6.12255300	0.67334800	0.33548400
C	-5.05779100	-2.08220700	-0.97060000
H	-4.58075000	-2.39545700	-1.90136800
H	-4.64698900	-2.67125000	-0.14829500
H	-6.12004400	-2.28867000	-1.04333200
C	5.32897500	-2.22790000	-0.50241100
H	4.84532600	-2.81537500	0.28087100

H	4.91161400	-2.51813900	-1.46861500
H	6.38787000	-2.46214700	-0.50276700
C	6.35577900	0.02011300	-0.05835100
H	6.45542200	0.78039900	-0.83521200
H	6.33409600	0.51013900	0.91629900
H	7.22763300	-0.62378700	-0.09633900

- **(MB-Cl-W)$_2$ (ωB97XD/def2-TZVP in water solvent)**

0 1

C	-2.20659700	2.27881600	1.20723100
C	-1.24254200	1.34569600	1.68729500
C	0.07684600	1.46081800	1.35668200
C	0.53925000	2.50480700	0.46790700
C	-0.45046300	3.43199600	-0.00510400
C	-1.74278000	3.34308900	0.34924300
C	2.79742900	1.87395400	0.42023200
C	2.69733800	0.76165100	1.29152500
C	3.79734400	-0.01778700	1.58889600
H	3.67152000	-0.86181100	2.25125200
C	5.05571500	0.27301400	1.02948100
C	5.15785700	1.40052900	0.16078800
C	4.06980200	2.15998900	-0.12455100
H	-1.55629700	0.53934600	2.33519900
H	-0.12124900	4.21011700	-0.68044000
H	-2.44687300	4.06433200	-0.03817900
H	6.10463700	1.66267000	-0.28491800
H	4.15944700	3.00695400	-0.79236600
N	6.12996800	-0.50112100	1.27855300
N	1.77107300	2.66711600	0.06402700
S	1.19761000	0.34340300	2.04249100
N	-3.48334900	2.17514600	1.51533900
Cl	-3.93825600	-1.21079500	3.66112600

O	2.13789800	4.35352800	-2.19446300
H	2.07335300	3.91179600	-1.32679400
H	2.03648500	5.29377900	-2.03343900
C	-4.23101200	-0.27429800	-0.95228700
C	-3.24222500	-1.16588200	-0.47886400
C	-1.92356500	-0.99343700	-0.80310800
C	-1.48766900	0.11249700	-1.61444900
C	-2.50437000	1.01089100	-2.07124800
C	-3.80721300	0.82983000	-1.77410600
C	0.79141000	-0.43056800	-1.65670900
C	0.70987000	-1.61280000	-0.86928600
C	1.82609200	-2.37227900	-0.59909600
H	1.71285400	-3.25759500	0.00750300
C	3.08742500	-2.01282300	-1.11451800
C	3.17239400	-0.82781600	-1.91155200
C	2.07310900	-0.07427400	-2.15474200
H	-3.52788700	-2.01400200	0.12417200
H	-2.18862100	1.85132400	-2.67610800
H	-4.53391900	1.53152000	-2.15452500
H	4.12035500	-0.51583500	-2.32162800
H	2.14835600	0.82466100	-2.75296500
N	4.17606600	-2.75806100	-0.86790600
N	-0.24367800	0.35080900	-1.97427400
S	-0.79124100	-2.14329000	-0.20816300
N	-5.51203400	-0.44241100	-0.64508500
Cl	-5.07488900	-4.43900800	-1.27454700
O	0.07763100	2.89459400	-3.49764700
H	0.06678400	2.07041700	-2.98923600
H	0.75267600	3.45259700	-3.08045600
C	-5.92391900	-1.57075600	0.17648800
H	-5.64711000	-2.50588200	-0.29658900
H	-5.47506200	-1.51881300	1.17156600

H	-7.00074800	-1.54919100	0.29666600
C	-6.54038800	0.43437000	-1.18546100
H	-6.37668000	1.47255800	-0.89248000
H	-6.57590500	0.37454500	-2.27488900
H	-7.50384500	0.12756200	-0.79582800
C	4.08286200	-3.90798300	0.01121800
H	3.39301800	-4.65988300	-0.37764400
H	5.06228700	-4.36629200	0.10705400
H	3.73989400	-3.60372800	1.00374500
C	5.48512200	-2.35383200	-1.36090700
H	5.51522900	-2.29616000	-2.44876100
H	5.76752800	-1.39347400	-0.92108100
H	6.21498100	-3.09029700	-1.03892400
C	6.01314300	-1.65266400	2.15269000
H	6.97749600	-2.14674300	2.21501400
H	5.70900100	-1.36471500	3.16144200
H	5.28923400	-2.37213400	1.76706800
C	7.41164400	-0.19819200	0.67018700
H	7.34958800	-0.25574300	-0.42536600
H	7.77204100	0.79309200	0.95165400
H	8.14195300	-0.93159900	0.99753900
C	-3.96778000	1.01258600	2.33134700
H	-3.74646100	0.07117000	1.84892300
H	-3.48853000	0.93782700	3.29535600
H	-5.03615300	1.13757200	2.44950000
C	-4.45365500	3.15687000	1.06975400
H	-4.21428900	4.14991700	1.45354500
H	-4.49507800	3.20436100	-0.02019100
H	-5.43585000	2.87923700	1.43696400
